# A pyruvate carbon flux tugging strategy for increasing 2,3-butanediol production and reducing ethanol subgeneration in the yeast *Saccharomyces cerevisiae*

**DOI:** 10.1186/s13068-018-1176-y

**Published:** 2018-06-26

**Authors:** Jun Ishii, Keisuke Morita, Kengo Ida, Hiroko Kato, Shohei Kinoshita, Shoko Hataya, Hiroshi Shimizu, Akihiko Kondo, Fumio Matsuda

**Affiliations:** 1 Graduate School of Science, Technology and Innovation, Kobe University, 1-1 Rokkodai, Nada, Kobe, 657-8501 Japan; 2 Department of Bioinformatic Engineering, Graduate School of Information Science and Technology, Osaka University, 1-5 Yamadaoka, Suita, Osaka 565-0871 Japan; 3 Department of Chemical Science and Engineering, Graduate School of Engineering, Kobe University, 1-1 Rokkodai, Nada, Kobe, 657-8501 Japan; 4 RIKEN Center for Sustainable Resource Science, 1-7-22 Suehiro, Tsurumi, Yokohama, 230-0045 Japan

**Keywords:** Pyruvate flux, Crabtree effect, Acetolactate synthase, Ethanol subgeneration, 2,3-Butanediol production, Pyruvate decarboxylase (PDC) deficient

## Abstract

**Background:**

The yeast *Saccharomyces cerevisiae* is a promising host cell for producing a wide range of chemicals. However, attempts to metabolically engineer Crabtree-positive *S. cerevisiae* invariably face a common issue: how to reduce dominant ethanol production. Here, we propose a yeast metabolic engineering strategy for decreasing ethanol subgeneration involving tugging the carbon flux at an important hub branching point (e.g., pyruvate). Tugging flux at a central glycolytic overflow metabolism point arising from high glycolytic activity may substantially increase higher alcohol production in *S. cerevisiae*. We validated this possibility by testing 2,3-butanediol (2,3-BDO) production, which is routed via pyruvate as the important hub compound.

**Results:**

By searching for high-activity acetolactate synthase (ALS) enzymes that catalyze the important first-step reaction in 2,3-BDO biosynthesis, and tuning several fermentation conditions, we demonstrated that a stronger pyruvate pulling effect (tugging of pyruvate carbon flux) is very effective for increasing 2,3-BDO production and reducing ethanol subgeneration by *S. cerevisiae*. To further confirm the validity of the pyruvate carbon flux tugging strategy, we constructed an evolved pyruvate decarboxylase (PDC)-deficient yeast (PDCΔ) strain that lacked three isozymes of PDC. In parallel with re-sequencing to identify genomic mutations, liquid chromatography–tandem mass spectrometry analysis of intermediate metabolites revealed significant accumulation of pyruvate and NADH in the evolved PDCΔ strain. Harnessing the high-activity ALS and additional downstream enzymes in the evolved PDCΔ strain resulted in a high yield of 2,3-BDO (a maximum of 0.41 g g^−1^ glucose consumed) and no ethanol subgeneration, thereby confirming the utility of our strategy. Using this engineered strain, we demonstrated a high 2,3-BDO titer (81.0 g L^−1^) in a fed-batch fermentation using a high concentration of glucose as the sole carbon source.

**Conclusions:**

We demonstrated that the pyruvate carbon flux tugging strategy is very effective for increasing 2,3-BDO production and decreasing ethanol subgeneration in Crabtree-positive *S. cerevisiae*. High activity of the common first-step enzyme for the conversion of pyruvate, which links to both the TCA cycle and amino acid biosynthesis, is likely important for the production of various chemicals by *S. cerevisiae*.

**Electronic supplementary material:**

The online version of this article (10.1186/s13068-018-1176-y) contains supplementary material, which is available to authorized users.

## Background

**Fig. 1:**
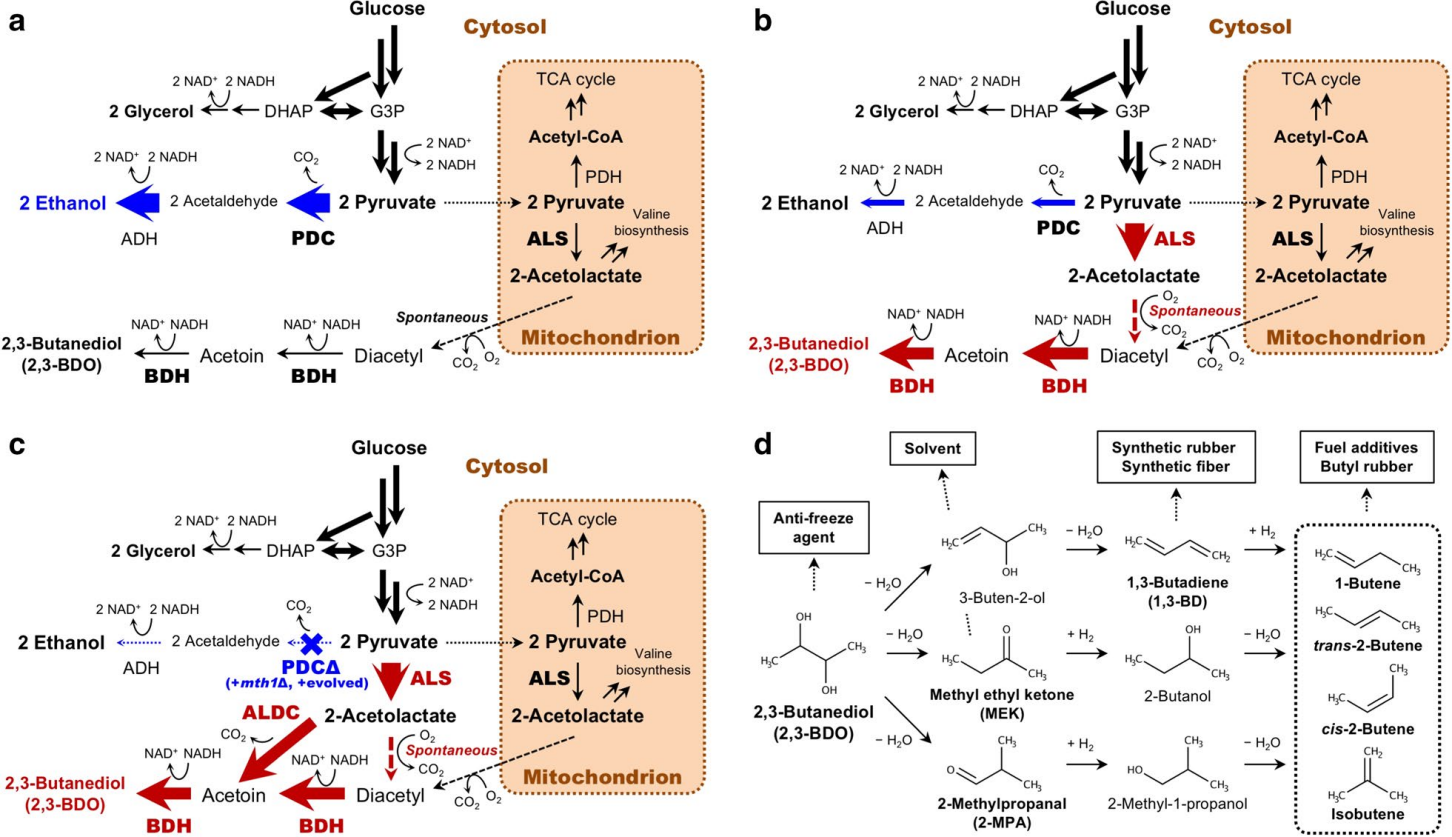
Pyruvate carbon flux tugging strategies for increased 2,3-butanediol (2,3-BDO) production and reduced ethanol subgeneration in yeast. **a** Ethanol and 2,3-BDO biosynthetic pathways in the wild-type yeast *S. cerevisiae*. Yeast preferentially produces ethanol even under aerobic conditions via the “Crabtree effect”, which remains to be completely elucidated. However, the strong activities of the glycolytic Embden–Meyerhof–Parnas (EMP) pathway and fermentative ethanol biosynthetic pathway in maintaining the redox homeostasis of NADH likely contribute to the Crabtree effect. Wild-type *S. cerevisiae* produces a small amount of 2,3-BDO via 2-acetolactate synthesized in the mitochondria. **b** Metabolic engineering strategy to tug the pyruvate carbon flux in yeast. High-activity cytosolic (or mitochondrial) acetolactate synthase (ALS) is required for increased 2,3-BDO production and reduced ethanol subgeneration. **c** A pyruvate decarboxylase (PDC)-deficient yeast (PDCΔ) strain (containing the *MTH1*-Δ*T* allele and subjected to laboratory evolution) was used to further ensure tugging of the pyruvate carbon flux and secure higher 2,3-BDO production. Acetolactate decarboxylase (ALDC) and butanediol dehydrogenase (BDH) were additionally (over)expressed to avoid clogging the carbon flux toward 2,3-BDO biosynthesis. **d** Applications of 2,3-BDO and its derivatives. 2,3-BDO can be chemically converted to various chemicals, including synthetic rubbers and fuel additives. *G3P* glyceraldehyde 3-phosphate, *DHAP* dihydroxyacetone phosphate, *TCA* tricarboxylic acid, *ADH* alcohol dehydrogenase, *PDH* pyruvate dehydrogenase

The budding (brewer’s or baker’s) yeast *Saccharomyces cerevisiae* is a traditional microorganism used for various industrial applications. Historically, *S. cerevisiae* has mainly been used for alcohol (ethanol) fermentations (e.g., beer and wine) [1], but the use of this yeast as a microbial cell factory was recently proposed for producing a wide range of chemicals including higher alcohols (e.g., linear or branched alcohols and diols, such as 1-propanol [2], *n*-butanol [3], isobutanol [4] and 1,3-propanediol [5]). To date, the focus has mainly been on the production of 2,3-butanediol (2,3-BDO) [6–9] because it is relatively easy to engineer high-producing yeast strains due to their redox balanced NADH-dependent reductive reaction(s) (Fig. 1a–c) [10], the low toxicity of 2,3-BDO to microbes [11]), and the wide applicability of 2,3-BDO as a platform chemical (Fig. 1d).

2,3-BDO can be directly used as an anti-freeze agent or chemically converted to other chemicals (Fig. 1d) [10, 12]. For example, 1,3-butadiene (1,3-BD) can be produced by the two-step dehydration of 2,3-BDO [13, 14]. 1,3-BD is a feedstock for the generation of synthetic rubber [15], and for the synthesis of hexamethylene diamine and adipic acid [16]. 2,3-BDO can also be converted to methyl ethyl ketone (MEK; butanone), 2-methylpropanal (2-MPA; isobutyraldehyde), and several butene derivatives by dehydration and/or hydrogenation (Fig. 1d). Thus, 2,3-BDO is a promising starting material for the synthesis of various compounds, including resin solvents, fuel additives, and butyl rubber (Fig. 1d) [13, 14, 17, 18].

The metabolic engineering of *S. cerevisiae* to produce various chemicals generally faces a common challenge: how to reduce dominant ethanol production [19]. In the presence of a high concentration of glucose, *S. cerevisiae* produces ethanol dominantly, even under aerobic conditions, due to a process known as the “Crabtree effect” (Fig. 1a) [20, 21]. Increasing the concentration of glucose reduces the need for oxidative phosphorylation by the tricarboxylic acid (TCA) cycle and accelerates glycolysis as the major source of energy. Although the mechanism of this glycolytic overflow metabolism is not completely understood, it is assumed to involve the redox homeostasis of NADH between the glycolytic and fermentative (ethanol) pathways [20, 21], in which a net of two NADH molecules are generated from one glucose molecule via glycolysis and subsequently oxidized in the ethanol formation reaction [22]) (Fig. 1a). It has been experimentally demonstrated that increased NADH oxidation can reduce ethanol formation [23]. Additionally, an in silico metabolic simulation suggested that the greater difficulty in metabolically engineering the eukaryote *S. cerevisiae* to produce higher alcohol concentrations compared to the prokaryote *Escherichia coli* is due to the structurally limited flexibility of the central metabolism and mitochondrial compartmentation of eukaryotes [24].

**Fig. 2:**
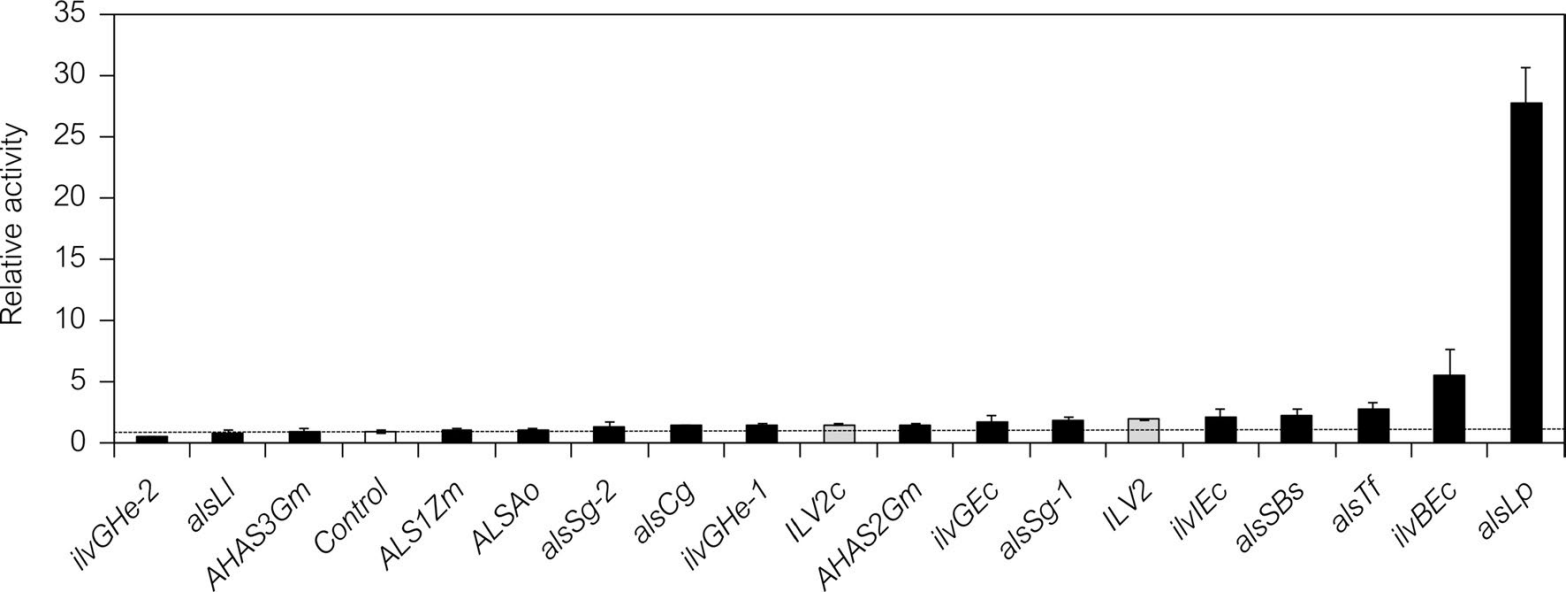
Relative enzymatic activities of acetolactate synthase (ALS) in crude yeast cell extracts. Various ALS (*ilv* and *als*) genes were expressed in *S. cerevisiae* YPH499 strain (YIDB001–019). Crude protein extracts were prepared from cells grown in 5 mL SD selection medium for 48 h and used to measure relative ALS enzyme activities (see Additional file 3: Method S6). The white bar shows the relative ALS activity of the control strain harboring a mock control vector (pGK425; YIDB001) and was used for normalization. The gray bars show the relative ALS activities of yeast strains expressing the *ILV2* gene (encoding the endogenous *S. cerevisiae* ALS; YIDB002) and the *ILV2c* gene (a truncated version of the *ILV2* gene lacking the mitochondrial signal sequence to localize Ilv2p in the cytosol; YIDB003) as positive controls. The black bars show the relative ALS activities of yeast strains expressing various ALS genes (YIDB004–019). Ao, *Aspergillus oryzae*; Bs, *Bacillus subtilis*; Cg, *Corynebacterium glutamicum*; Ec, *Escherichia coli*; Gm, *Glycine max*; He, *Halomonas elongata*; Ll, *Lactococcus lactis*; Lp, *Lactobacillus plantarum*; Sg, *Streptomyces griseus*; Tf, *Thermobifida fusca*; Zm, *Zea mays*. Data are presented as the mean ± standard deviation of three independent transformants (*n* = 3 each)

The NADH-dependent reductive reaction(s) may provide a strategy for using the 2,3-BDO biosynthesis pathway to displace the ethanol biosynthesis pathway (Fig. 1a) [10, 19]. In wild-type *S. cerevisiae*, 2-acetolactate is synthesized by the endogenous acetolactate synthase (ALS) Ilv2p, which localizes to the mitochondria and catalyzes the conversion of pyruvate (Fig. 1a) [12, 25]. Spontaneous decarboxylation can convert 2-acetolactate into diacetyl in the presence of oxygen. Next, diacetyl can be converted in the yeast cytosol into acetoin and 2,3-BDO via the NADH-dependent two-step reductive reactions of butanediol dehydrogenase (BDH; EC 1.1.1.4), an enzyme that also has diacetyl reductase activity and is encoded by the *BDH1* (or *BDH2*) gene in *S. cerevisiae* (Fig. 1a) [12]. In contrast, many bacteria synthesize 2-acetolactate in the cytosol (Fig. 1b) and then convert it into acetoin via two routes: (1) a two-step conversion by spontaneous decarboxylation and an NADH-dependent diacetyl reductase reaction (Fig. 1b), or (2) an enzymatic one-step conversion by acetolactate decarboxylase (ALDC) (Fig. 1c) [12]. Similar to *S. cerevisiae*, bacteria also further convert acetoin into 2,3-BDO by an NADH-dependent BDH reductive reaction [12]. Although redox balance is not completely maintained in the bacterial ALDC reaction, moderate production of 2,3-BDO is possible.

To achieve increased production of higher alcohols and reduced subgeneration of ethanol in Crabtree-positive *S. cerevisiae*, in this study we investigated the utility of tugging high glycolytic carbon flux into 2,3-BDO biosynthesis by introducing a stronger pulling effect at the hub branching point (pyruvate) (Fig. 1b). For this purpose, we searched gene databases for a high-activity ALS enzyme and confirmed the effectiveness of the identified enzyme for increasing 2,3-BDO production and reducing ethanol subgeneration. We demonstrated the validity of our strategy by constructing a pyruvate decarboxylase (PDC)-deficient yeast (PDCΔ) strain and conducted experimental evolution to improve its growth rate (Fig. 1c). In parallel with genome re-sequencing for identifying mutations, we conducted liquid chromatography–tandem mass spectrometry (LC–MS/MS) analysis of the intermediate metabolites. This analysis revealed significant accumulation of pyruvate and NADH in the evolved PDCΔ strain. The combination of the high-activity ALS and the manipulation of additional downstream enzymes for 2,3-BDO biosynthesis into this evolved PDCΔ strain provided a high yield of 2,3-BDO (a maximum of 0.41 g g^−1^ glucose consumed, the highest value reported to date for *S. cerevisiae* [12, 26]). Using this engineered strain, we tuned the fermentation conditions and achieved a quite high 2,3-BDO titer (81.0 g L^−1^) in fed-batch fermentations using a high concentration of glucose as the sole carbon source.

## Methods

### Yeast strains and media

Details of the *S. cerevisiae* strain YPH499 [27] (Stratagene/Agilent Technologies, Palo Alto, CA, USA) and other recombinant strains used in this study and their genotypes are outlined in Table 1.

**Table 1:** Yeast strains used in this study

Strain	Genotypes	Source
YPH499	*MAT***a** *ura3*-*52 lys2*-*801 ade2*-*101 trp1*-Δ*63 his3*-Δ*200 leu2*-Δ*1*	[27]
YJI2	YPH499 *pdc5*Δ	This study
YJI6	YPH499 *pdc5*Δ *pdc6*Δ	This study
YSM009	YPH499 *pdc5*Δ *pdc6*Δ *MTH1*-Δ*T* (genome sequence was not determined)	This study
YSM021 (PDCΔ)	YPH499 *pdc1*Δ *pdc5*Δ *pdc6*Δ *MTH1*-Δ*T(L165F)*	This study
Evo44	Obtained from YSM021 by laboratory evolution in SD medium (culture series 1) for 44 days	This study
Evo44-1	Colony #1 isolated from Evo44 glycerol stock	This study
Evo44-2	Colony #2 isolated from Evo44 glycerol stock	This study
Evo44-3	Colony #3 isolated from Evo44 glycerol stock	This study
Evo122	Obtained from YSM021 by laboratory evolution in SD medium (culture series 1) for 122 days	This study
Evo122-1	Colony #1 isolated from Evo122 glycerol stock	This study
Evo122-2	Colony #2 isolated from Evo122 glycerol stock	This study
Evo122-3	Colony #3 isolated from Evo122 glycerol stock	This study
YSM046 (PDCΔ + evolved)	Identical to Evo122-2: a laboratory-evolved yeast strain derived from PDCΔ (YSM021) strain	This study

Full details of the culture media are described in Additional file 3: Method S1. Briefly, yeast–peptone–dextrose–adenine (YPDA), minimal synthetic dextrose (SD), and synthetic complete (SC) media containing 20 g L^−1^ glucose were used. For fermentation tests, SD medium contained a high concentration of glucose (25, 50 or 100 g L^−1^) as the sole carbon source. Amino acids and nucleotides were supplemented as necessary because SD selection medium lacks these auxotrophic components.

### Yeast strain construction

Full details of the construction of the gene deletion mutants are described in Additional file 3: Method S2 and are shown in Additional file 1: Fig. S1. All yeast strains used in this study were generated from the YPH499 parental strain [27] and are listed in Table 1. All primers used for the construction of the yeast gene deletion mutants are listed in Additional file 2: Table S1. Using YPH499 as the parental strain, a quadruple deletion mutant (*MTH1*-Δ*T pdc1*Δ *pdc5*Δ *pdc6*Δ) YSM021 strain (PDCΔ strain) (Table 1) was constructed using the seamless marker recycling method (URA-blaster method) [28].

### Plasmid construction and yeast transformation

Full details regarding plasmid construction and yeast transformation are described in Additional file 3: Method S3. All plasmids used in this study are listed in Table 2. All primers used for plasmid construction are listed in Additional file 2: Table S1. The transformation of plasmid DNA was carried out using the lithium acetate method [29]. All transformants generated in this study are listed in Table 3.

**Table 2:** Plasmids used in this study

Plasmid	Specific features	Source
Vectors		
pGK425	Yeast multi-copy type single-gene expression vector containing *PGK1* promoter, *PGK1* terminator, 2*μ* origin, and *LEU2* marker	[37]
pAT425	Yeast multi-copy type two-gene expression vector containing *ADH1* and *TDH3* promoters and terminators, 2*μ* origin, *LEU2* marker	[39]
pATP422	Yeast multi-copy type three-gene expression vector containing *ADH1*, *TDH3* and *PGK1* promoters and terminators, 2*μ* origin, *ADE2* marker	[39]
Yeast acetolactate synthase (ALS) expressions		
pGK425-ILV2	pGK425, expression of *Saccharomyces cerevisiae* ALS (*ILV2*) gene	[4]
pGK425-ILV2c	pGK425, expression of truncated version of *S. cerevisiae* ALS lacking the N-terminal (mitochondrial) signal sequence (*ILV2c*) gene	[38]
ALS expressions		
pGK425-ALSAo	pGK425, expression of *Aspergillus oryzae* ALS (*ALS* or *AO090009000123*) gene	This study
pGK425-alsSBs	pGK425, expression of *Bacillus subtilis* ALS (*alsS*) gene	This study
pGK425-alsCg	pGK425, expression of *Corynebacterium glutamicum* ALS (*als* or *Cgl1271*) gene	This study
pGK425-ilvBEc	pGK425, expression of *Escherichia coli* ALS (*ilvB*) gene	This study
pGK425-ilvGEc	pGK425, expression of *E. coli* ALS (*ilvG*) gene	This study
pGK425-ilvIEc	pGK425, expression of *E. coli* ALS (*ilvI*) gene	This study
pGK425-AHAS2Gm	pGK425, expression of *Glycine max* ALS (*AHAS2*) gene	This study
pGK425-AHAS3Gm	pGK425, expression of *G. max* ALS (*AHAS3*) gene	This study
pGK425-ilvGHe-1	pGK425, expression of *Halomonas elongata* ALS (*ilvG*) gene 1 [HELO_1296]	This study
pGK425-ilvGHe-2	pGK425, expression of *H. elongata* ALS (*ilvG*) gene 2 [HELO_2761]	This study
pGK425-alsLl	pGK425, expression of *Lactococcus lactis* ALS (*als*) gene	This study
pGK425-alsLp	pGK425, expression of *Lactobacillus plantarum* ALS (*als*) gene	This study
pGK425-alsSg-1	pGK425, expression of *Streptomyces griseus* ALS (*als*) gene 1 [SGR_4789]	This study
pGK425-alsSg-2	pGK425, expression of *S. griseus* ALS (*als*) gene 2 [SGR_6206]	This study
pGK425-alsTf	pGK425, expression of *Thermobifida fusca* ALS (*als*) gene [Tfu_0611]	This study
pGK425-ALS1Zm	pGK425, expression of *Zea mays* ALS (*ALS1*) gene [100285396]	This study
High-activity ALS expressions (codon optimized)		
pGK425-ilvBEcOp	pGK425, expression of *E. coli* codon-optimized *ilvB* (*ilvBEcOp*) gene	This study
pGK425-alsLpOp	pGK425, expression of *L. plantarum* codon-optimized *als* (*alsLpOp*) gene	This study
pGK425-alsTfOp	pGK425, expression of *T. fusca* codon-optimized *als* (*alsTfOp*) gene	This study
pATP422-alsLpOp	pATP422, expression of *alsLpOp* gene by *PGK1* promoter	This study
Acetolactate decarboxylase (ALDC) expressions (codon optimized)		
pATP422-alsLpOp-aldcBsOp	pATP422, expressions of *alsLpOp* gene by *PGK1* promoter and *B. subtilis* codon-optimized ALDC (*aldcBsOp*) gene by *TDH3* promoter	This study
pATP422-alsLpOp-aldcEaOp	pATP422, expressions of *alsLpOp* gene by *PGK1* promoter and *Enterobacter aerogenes* codon-optimized ALDC (*aldcEaOp*) gene by *TDH3* promoter	This study
pATP422-alsLpOp-aldcKpOp	pATP422, expressions of *alsLpOp* gene by *PGK1* promoter and *Klebsiella pneumoniae* codon-optimized ALDC (*aldcKpOp*) gene by *TDH3* promoter	This study
pATP422-alsLpOp-aldcLlOp	pATP422, expressions of *alsLpOp* gene by *PGK1* promoter and *L. lactis* codon-optimized ALDC (*aldcLlOp*) gene by *TDH3* promoter	This study
Yeast butanediol dehydrogenase (BDH) expression		
pATP422-alsLpOp-BDH1	pATP422, expressions of *alsLpOp* gene by *PGK1* promoter and *S. cerevisiae* BDH (*BDH1*) gene by *ADH1* promoter	This study
pAT425-BDH1	pAT425, expressions of *S. cerevisiae* BDH (*BDH1*) gene by *ADH1* promoter	This study

**Table 3:** Yeast transformants used in this study

Transformant	Host strain	Plasmid	Gene 1 (ALS)	Gene 2 (ALDC)	Gene 3 (BDH)
YIDB001	YPH499	pGK425	–	–	–
YIDB002	YPH499	pGK425-ILV2	*ILV2* (*S. cerevisiae*)	–	–
YIDB003	YPH499	pGK425-ILV2c	*ILV2c* (*S. cerevisiae*)	–	–
YIDB004	YPH499	pGK425-ALSAo	*ALSAo* (*A. oryzae*)	–	–
YIDB005	YPH499	pGK425-alsSBs	*alsSBs* (*B. subtilis*)	–	–
YIDB006	YPH499	pGK425-alsCg	*alsCg* (*C. glutamicum*)	–	–
YIDB007	YPH499	pGK425-ilvBEc	*ilvBEc* (*E. coli*)	–	–
YIDB008	YPH499	pGK425-ilvGEc	*ilvGEc* (*E. coli*)	–	–
YIDB009	YPH499	pGK425-ilvIEc	*ilvIEc* (*E. coli*)	–	–
YIDB010	YPH499	pGK425-AHAS2Gm	*AHAS2Gm* (*G. max*)	–	–
YIDB011	YPH499	pGK425-AHAS3Gm	*AHAS3* (*G. max*)	–	–
YIDB012	YPH499	pGK425-ilvGHe-1	*ilvGHe*-*1* (*H. elongata*)	–	–
YIDB013	YPH499	pGK425-ilvGHe-2	*ilvGHe*-*2* (*H. elongata*)	–	–
YIDB014	YPH499	pGK425-alsLl	*alsLl* (*L. lactis*)	–	–
YIDB015	YPH499	pGK425-alsLp	*alsLp* (*L. plantarum*)	–	–
YIDB016	YPH499	pGK425-alsSg-1	*alsSg*-*1* (*S. griseus*)	–	–
YIDB017	YPH499	pGK425-alsSg-2	*alsSg*-*2* (*S. griseus*)	–	–
YIDB018	YPH499	pGK425-alsTf	*alsTf* (*T. fusca*)	–	–
YIDB019	YPH499	pGK425-ALS1Zm	*ALSZm* (*Z. mays*)	–	–
YIDB022	YPH499	pGK425-ilvBEcOp	*ilvBEcOp* (*E. coli*, optimized)	–	–
YIDB023	YPH499	pGK425-alsLpOp	*alsLpOp* (*L. plantarum*, optimized)	–	–
YIDB024	YPH499	pGK425-alsTfOp	*alsTfOp* (*T. fusca*, optimized)	–	–
YIDB025	YPH499	pATP422-alsLpOp	*alsLpOp* (*L. plantarum*, optimized)	–	–
YIDB030	YPH499	pATP422-alsLpOp-aldcBsOp	*alsLpOp* (*L. plantarum*, optimized)	*aldcBsOp* (*B. subtilis*, optimized)	–
YIDB031	YPH499	pATP422-alsLpOp-aldcEaOp	*alsLpOp* (*L. plantarum*, optimized)	*aldcEaOp* (*E. aerogenes*, optimized)	–
YIDB032	YPH499	pATP422-alsLpOp-aldcKpOp	*alsLpOp* (*L. plantarum*, optimized)	*aldcKpOp* (*K. pneumoniae*, optimized)	–
YIDB033	YPH499	pATP422-alsLpOp-aldcLlOp	*alsLpOp* (*L. plantarum*, optimized)	*aldcLlOp* (*L. lactis*, optimized)	–
YIDB034	YPH499	pATP422-alsLpOp-BDH1	*alsLpOp* (*L. plantarum*, optimized)	–	*BDH1* (*S. cerevisiae*)
YHI010	YSM046	pGK425-alsLpOp	*alsLpOp* (*L. plantarum*, optimized)	–	–
YHI011	YSM046	pATP422-alsLpOp-aldcLlOp	*alsLpOp* (*L. plantarum*, optimized)	*aldcLlOp* (*L. lactis*, optimized)	–
YHI027	YPH499	pATP422-alsLpOp-aldcLlOp/pAT425-BDH1	*alsLpOp* (*L. plantarum*, optimized)	*aldcLlOp* (*L. lactis*, optimized)	*BDH1* (*S. cerevisiae*)
YHI030	YSM046	pATP422-alsLpOp-aldcLlOp/pAT425-BDH1	*alsLpOp* (*L. plantarum*, optimized)	*aldcLlOp* (*L. lactis*, optimized)	*BDH1* (*S. cerevisiae*)
YSHB001	YSM021	pATP422-alsLpOp-aldcLlOp/pAT425-BDH1	*alsLpOp* (*L. plantarum*, optimized)	*aldcLlOp* (*L. lactis*, optimized)	*BDH1* (*S. cerevisiae*)

### Laboratory evolution

Full details of the laboratory evolution experiments are described in Additional file 3: Method S4. For laboratory evolution of the PDCΔ (YSM021) strain (Table 1), a passage culture in SD medium was carried out with five independent culture series. Cells of culture series 1 at days 44 and 122 (Evo44 and Evo122 strains) (Table 1) were stored as glycerol stocks. An isolate of Evo122 (Evo122-2) was designated the YSM046 strain (evolved PDCΔ strain) (Table 1).

### Genome re-sequencing

Full details of the genome re-sequencing experiments are described in Additional file 3: Method S5. The whole genomes of YPH499, YSM021, and six evolved strains were sequenced by pair-end sequencing (250 bp) using an Illumina HiSeq2000 next-generation sequencer and a MiSeq reagent 500 cycle kit v2 (Illumina, San Diego, CA, USA), then analyzed with Bowtie2 [30], SAMtools [31], and Integrative Genomics Viewer (IGV) [32] software.

### Relative activity measurements of acetolactate synthase (ALS) enzymes

Full details of the ALS enzyme assays are described in Additional file 3: Method S6). Yeast cells were grown in SD selection medium and then the collected cells were disrupted with glass beads using a Shake Master Neo (Bio Medical Science, Tokyo, Japan) to obtain cell crude extracts. The ALS activity assay basically followed previously described procedures [33, 34]. Briefly, a pre-mixture containing thiamine pyrophosphate, FAD, MgCl_2_ and crude yeast cell extract was pre-incubated, and then the reaction was started by adding pyruvate. The reaction was stopped by adding sulfonic acid and the reaction mixture was incubated to convert 2-acetolactate to acetoin. Acetoin was then further oxidized to diacetyl with creatine and α-naphthol. The relative ALS activity based on the colorimetric assay of acetoin (or diacetyl) was determined by measuring the absorbance (at 525 nm) of the developed red color using an EnVision multilabel plate reader (Perkin Elmer, Waltham, MA, USA).

### Culture conditions

Full details of the culture conditions are described in Additional file 3: Method S7. Yeast cells were seeded at a low initial optical density (OD) and grown or used for fermentation in SD or SD selection media containing 20 g L^−1^ glucose at 30 °C. The fermentation conditions of the YHI030 strain (Table 2) were tuned by inoculating the pre-cultured cells into 50 mL of fresh SD selection medium (containing either 25, 50 or 100 g L^−1^ glucose) to give an initial cell density of 15 g L^−1^ (wet cell weight). Buffer medium was pH-adjusted by adding 2-(*N*-morpholino)ethanesulfonic acid (MES) to the SD selection medium. Additional glucose was added to the fermented culture for fed-batch fermentations.

### Analysis of extracellular metabolites

Full details of the analysis of extracellular metabolites are described in Additional file 3: Method S8. The concentrations of 2,3-BDO, glucose, ethanol, glycerol, acetoin, pyruvate, acetate, lactate and succinate in the culture medium were determined using a high-performance liquid chromatography (HPLC) system (with UV/Vis and refractive index detectors) (Shimadzu, Kyoto, Japan) equipped with an Aminex HPX-87H column (300 × 7.8 mm) (Bio-Rad, Hercules, CA, USA).

### LC–MS/MS analysis of intermediate metabolites

Full details of the LC–MS/MS analysis of the intermediate metabolites are described in Additional file 3: Method S9. Metabolite analysis was performed using a previously described method [35].

## Results

### Selection of acetolactate synthase (ALS) for 2,3-BDO biosynthesis

ALS is the first-step enzyme in valine biosynthesis, catalyzes the conversion of pyruvate to 2-acetolactate, and is important for 2,3-BDO biosynthesis (Fig. 1a, b) [4, 36]. To tug the carbon flux from pyruvate and enhance 2,3-BDO biosynthesis in *S. cerevisiae*, we isolated the genes encoding ALS enzymes from various species. Sixteen ALS genes from 11 species other than *S. cerevisiae* (12 genes were from eight prokaryotes and 4 genes were from three eukaryotes) were cloned into the pGK425 yeast expression vector [37] (Table 2) and expressed under the *PGK1* promoter in YPH499 (wild-type) yeast strain (Table 3). The relative ALS activities of crude cell extracts were measured after 48 h of cultivation (Fig. 2). *ILV2* (encoding the endogenous *S. cerevisiae* ALS [4]) and *ILV2c* (a truncated version of the *ILV2* gene lacking the mitochondrial signal sequence to localize Ilv2 in cytosol [38]) genes (Fig. 1a, b) were used as positive controls to assess the capabilities of the ALS enzymes. Most of the constructed strains showed higher ALS activities than the control strain (harboring the mock control vector) that inherently contains the *ILV2* gene in the genome (Fig. 2). Eight strains exhibited higher ALS activities than the strain expressing the truncated (cytosolic) *ILV2c* gene, and five strains displayed higher ALS activities than the strain overexpressing the intact (mitochondrial) *ILV2* gene. The relative measured ALS activities were used to identify the top three genes as *alsLp* (from *Lactobacillus plantarum*; 27.8-fold), *ilvBEc* (from *Escherichia coli*; 5.5-fold) and *alsTf* (from *Thermobifida fusca*; 2.8-fold). Notably, the *alsLp* gene encoded an enzyme with much higher ALS activity than any other ALS tested (Fig. 2).

**Fig. 3:**
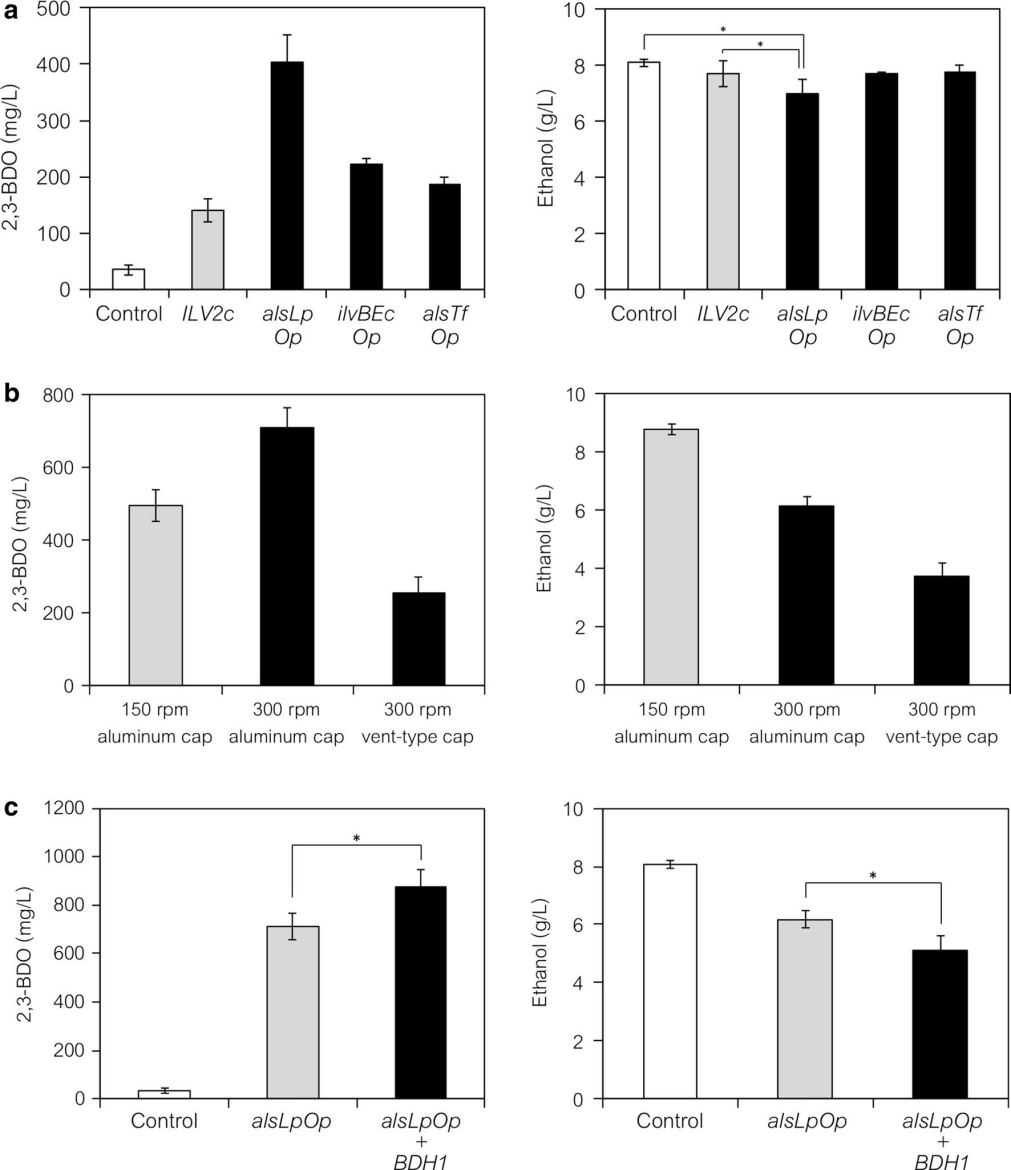
Investigation of genotype and aeration conditions for 2,3-BDO production by the YPH499 wild-type strain. Ec, *Escherichia coli*; Lp, *Lactobacillus plantarum*; Tf, *Thermobifida fusca*. Op, codon-optimized. Fermentations were performed in test tubes containing 3 mL of SD selection medium (20 g L^−1^ glucose) under the indicated aeration conditions. The concentrations of 2,3-BDO and ethanol in the media were determined at 48 h after the start of fermentation. **a** 2,3-BDO production by YPH499 strains expressing high-activity ALSs (black bars; YIDB022–025) under semi-aerobic conditions (150 rpm, aluminum cap). A truncated version of the *ILV2* gene (*ILV2c*), which is expressed in the cytosol, was used as a comparative yeast ALS (gray bars). YPH499 harboring the mock vector was used as the negative control (white bars). **b** Effect of aeration on 2,3-BDO production by YPH499 strain expressing high-activity ALS (*alsLpOp* gene; YIDB025). The agitation speed (150 rpm) was increased to 300 rpm and the cover cap on the test tube was changed from an aluminum cap to a vent-type cap to increase the aeration. The gray bars indicate the baseline (former) conditions (150 rpm, aluminum cap) and the black bars indicate the altered conditions (300 rpm, aluminum or vent-type cap). **c** 2,3-BDO production by YPH499 strains co-expressing high-activity ALS (*alsLpOp* gene) and yeast endogenous BDH (*BDH1* gene) (black bars; YIDB034) under aerobic conditions (300 rpm, aluminum cap). YPH499 expressing only the ALS (*alsLpOp*) gene was used as the comparative control (gray bars). The YPH499 wild-type strain was used as the negative control (white bars). Data are, respectively, presented as the mean ± standard deviation of three independent transformants (for **a** and **c**) and of three separate cultivations (for **b**) (*n* = 3 each). Statistical significance was assessed by the *t* test (*p < 0.05)

The top three ALS genes were introduced into yeast cells and 2,3-BDO fermentations were performed in test tubes (Fig. 3a). Strains harboring the mock vector and the *ILV2c* expression plasmid were, respectively, used as negative and positive controls. The *alsLp*, *ilvBEc* and *alsTf* genes were codon-optimized (*alsLpOp*, *ilvBEcOp* and *alsTfOp*), subcloned into the same pGK425 expression vector (Table 2), and introduced into YPH499 (Table 3). The obtained strains were cultured in test tubes with 3 mL of SD medium (containing 20 g L^−1^ glucose) under semi-aerobic conditions (Fig. 3a). The expression of *ILV2c* in YPH499 resulted in a four-time higher 2,3-BDO titer (141 mg L^−1^) compared to the negative control strain (35 mg L^−1^), and expression of the top three ALS genes further increased the titer of 2,3-BDO. The increased levels of 2,3-BDO correlated with the strength of the relative ALS activity (Fig. 2). YPH499 strain expressing the *alsLpOp* gene yielded 403 mg L^−1^ 2,3-BDO, while those expressing the *ilvBEcOp* or *alsTfOp* genes produced 223 and 188 mg L^−1^ 2,3-BDO (Fig. 3a), respectively. The strain expressing *alsLpOp* resulted in a slight decrease in ethanol concentration (7.0 g L^−1^) compared to the mock control strain (8.1 g L^−1^) and the strain expressing *ILV2c* (7.7 g L^−1^) (*p *< 0.05; Fig. 3a).

### Demonstration of the tugging of pyruvate flux by simple tuning using a high-activity ALS-expressing wild-type strain

We increased the titer of 2,3-BDO and reduced ethanol subgeneration by further tuning the wild-type yeast strain expressing a high-activity ALS (*alsLpOp*) by introducing the expression of additional genes (ALDC genes and *BDH1*) and altering the aeration conditions (Additional file 1: Fig. S2, Fig. 3b, c). Co-expression of the *alsLpOp* and ALDC (codon-optimized) genes from several bacteria using the pATP422 multiple gene expression vector [39] (Table 2) in YPH499 (Table 3; YIDB030–033) did not result in an increase in 2,3-BDO compared to the strain expressing only the *alsLpOp* gene (Additional file 1: Fig. S2). This decrease in productivity might be attributed to the reduced consumption of NADH in 2,3-BDO biosynthesis via the ALDC reaction (Fig. 1c). Briefly, the ALDC pathway does not use NADH to convert 2-acetolactate to acetoin (Fig. 1c), while the alternative diacetyl pathway uses NADH through the reaction of diacetyl reductase (encoded by *BDH1* in *S. cerevisiae*) (Fig. 1b). Consequently, the overexpression of ALDC might decrease carbon flux toward 2,3-BDO to consume excess NADH during ethanol biosynthesis. Indeed, the ALDC-expressing strains produced higher amounts of ethanol than the non-expressing strain (data not shown).

Culture conditions (aeration) were investigated by changing the agitation speed (150–300 rpm) and the cover cap on the test tubes (aluminum cap to a vent-type cap, SILICOSEN^®^) using the YPH499 strain expressing the ALS (*alsLpOp*) gene (Fig. 3b). Under the same conditions as used for the previous cultures (aluminum cap, 150 rpm), the strain produced 494 mg L^−1^ 2,3-BDO and 8.8 g L^−1^ ethanol. The titers of YPH499 expressing the ALS (*alsLpOp*) gene slightly differed from those shown in Fig. 3a, possibly due to differences between the vector backbones (pGK425 and pATP422 in Fig. 3a, b, respectively). As aeration increased (aluminum cap and vent-type cap, 300 rpm), significant decreases in ethanol production were observed (6.2 and 3.7 g L^−1^) (Fig. 3b). A moderate increase in aeration (aluminum cap, 300 rpm) resulted in a higher 2,3-BDO production titer (711 mg L^−1^), whereas a large increase in aeration (vent-type cap, 300 rpm) reduced the 2,3-BDO titer (256 mg L^−1^) (Fig. 3b). It is possible that excessive aeration (vent-type cap, 300 rpm) may have resulted in the redirection of pyruvate flux towards the TCA cycle, faster respiration, and/or the evaporation of ethanol. Regardless, YPH499 expressing the ALS (*alsLpOp*) gene under high aeration conditions produced ethanol titers consistent with previous reports [20, 21]. In contrast, conditions of reduced aeration (aluminum cap, 300 rpm) could have reduced the spontaneous decarboxylation required for converting 2-acetolactate to diacetyl because this reaction requires moderate concentrations of oxygen [12].

*BDH1*, which encodes endogenous yeast BDH, was overexpressed along with *alsLpOp* using the pATP422 multiple gene expression vector (Table 2) in YPH499 (Table 3; YIDB034), resulting in the 2,3-BDO titer increasing up to 874 mg L^−1^ (*p *< 0.05; Fig. 3c). The titer of ethanol was reduced to 5.1 g L^−1^ (*p *< 0.05; Fig. 3c) because the carbon flux to 2,3-BDO biosynthesis was further enhanced by the additional expression of Bdh1 enzyme (Fig. 1b). These results demonstrated the validity of the pyruvate carbon flux strategy for 2,3-BDO production in the wild-type strain using high-activity ALS (the *alsLpOp* gene). However, this engineered strain (expressing the *alsLpOp* and *BDH1* genes) still exhibited a high titer of ethanol. Consequently, the disruption of ethanol biosynthesis is a strategy for tugging pyruvate carbon flux and further improving 2,3-BDO production.

### Construction of a pyruvate decarboxylase-deficient (PDCΔ) strain (YSM021 strain)

**Fig. 4:**
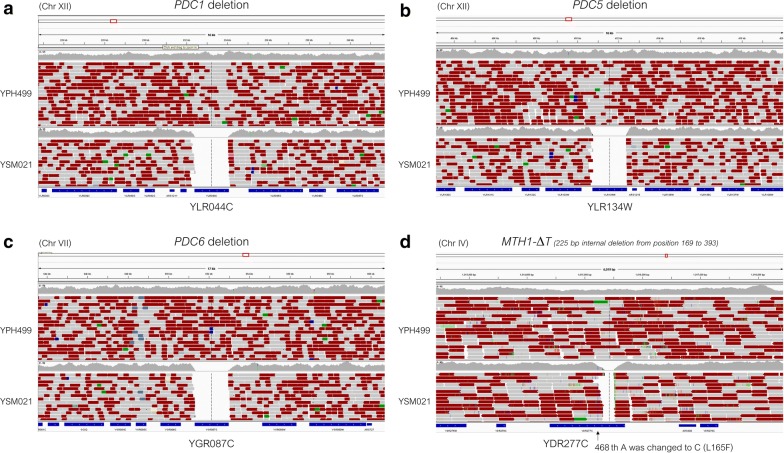
Deletion of genome regions in the PDCΔ (YSM021) strain. Deletion of the coding DNA sequence (CDS) regions of *PDC1* (**a**), *PDC5* (**b**), *PDC6* (**c**) and the internal deletion of *MTH1* (*MTH1*-Δ*T*) (**d**) was confirmed by genome sequencing (pair-end sequencing using Illumina MiSeq and Miseq v2 500 cycle kits). The data were processed and displayed using Integrative Genomic Viewer version 2.3.81

Full details of the results for constructing the PDCΔ (YSM021) strain are described in Additional file 3: Result S1. Briefly, we disrupted ethanol biosynthesis by following the strategy used to construct the previously reported pyruvate decarboxylase-deficient Pdc^−^ strain (deletion of the coding DNA sequence (CDS) regions of the *PDC1*, *PDC5* and *PDC6* genes, and introduction of the *MTH1*-Δ*T* allele) [40]. We constructed the pyruvate decarboxylase-deficient PDCΔ strain YSM021 (Table 1) from YPH499, and confirmed quadruple gene deletion (*MTH1*-Δ*T pdc1*Δ *pdc5*Δ *pdc6*Δ) by genome re-sequencing (Fig. 4a–d). The genome sequence of YSM021 strain further revealed the occurrence of a nonsynonymous substitution (L165F) in the *MTH1*-Δ*T* gene due to a single nucleotide polymorphism (SNP) located at position 468 (from A to C) (Fig. 4d, Table 1). Although the glucose consumption of YSM021 was much slower than that of YPH499, no ethanol, glycerol, or acetate production was detected in YSM021 cultures after 120 h of cultivation (Additional file 1: Fig. S3) but pyruvate accumulated with time (Additional file 1: Fig. S3).

### Laboratory evolution of the PDCΔ strain and genome re-sequencing

**Fig. 5:**
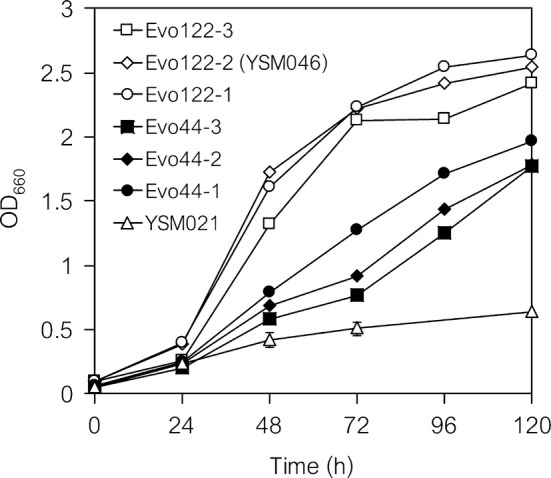
Growth of evolved PDCΔ strains. Three colonies isolated from Evo44 and three from Evo122 were cultured in 5 mL of SD medium under semi-aerobic conditions. An isolate of Evo122 (Evo122-2) was designated as YSM046 and used in additional experiments

Full details of the laboratory evolution of the PDCΔ (YSM021) strain and genome re-sequencing are described in Additional file 3: Result S2. Briefly, laboratory evolution of the PDCΔ (YSM021) strain using a series of five independent cultures allowed for faster growth of culture series 1 (Additional file 1: Fig. S4). Three colonies isolated from the cells collected at days 44 and 122 (Evo44 and Evo122) (Table 1) showed improved cell growth phenotypes (Fig. 5).

Genome re-sequencing revealed several nonsynonymous mutations, including an A to T mutation at position 1547 (D516V) in the *YAK1* gene (YJL141C) and a G to A mutation at position 605 (G202D) in the *MCT1* gene (YOR221C) (Table 4). *MCT1* is predicted to encode a malonyl-CoA:ACP transferase responsible for lipid biosynthesis [41]. *YAK1* encodes a serine–threonine protein kinase that inhibits growth in response to glucose availability in a glucose-sensing system [42, 43], suggesting that the mutation in *YAK1* is likely responsible for the observed improved cell growth rate. Further genetic characterization of these mutations (including L165F in the *MTH1*-Δ*T* gene) is in progress and will be reported elsewhere in the future.

**Table 4:** Mutations commonly observed in isolates of the evolved strain

Chromosome	Coordinate	ORFs	Position (bp)	Mutation	Type	Evo44	Evo122
Chr 2	27308	YBL101C (*ECM21*)	991	T → – (deletion)	Frame shift	nd	+
Chr 3	253645	YCR079W (*PTC6*)	804	C → T	Synonymous	nd	+
Chr 5	288490	tS(UGA)E (*SUP19*)	35	G → C	–	nd	+
Chr 10	148840	YJL141C (*YAK1*)	1547	A → T (D516V)	Nonsynonymous	+	+
Chr 15	756954	YOR221C (*MCT1*)	605	G → A (G202D)	Nonsynonymous	+	+

*nd* not detected

An isolate of Evo122 (Evo122-2) was designated as YSM046 (Table 1) and used for 2,3-BDO production. A comparison of culture profiles indicated that the cell growth rate of YSM046 strain was significantly improved compared to YSM021, and ethanol and glycerol were not produced (Additional file 1: Fig. S3).

### Metabolic state of the evolved PDCΔ strain (YSM046 strain)

**Fig. 6:**
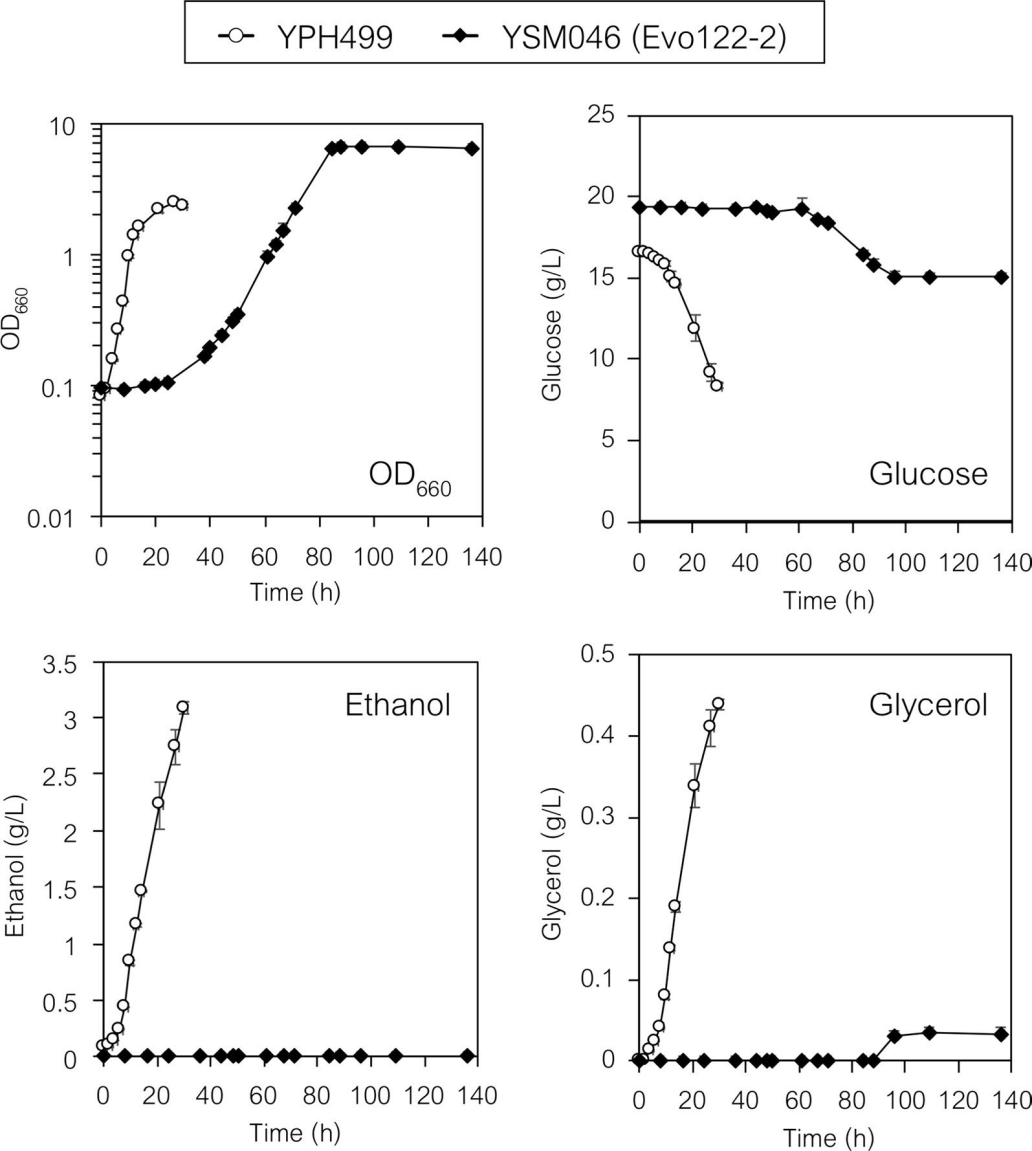
Flask-scale cultivation of YPH499 and YSM046 (evolved PDCΔ) strains under aerobic conditions. Cells were cultured in Sakaguchi flasks containing 100 mL of SD medium (20 g L^−1^ glucose). Data are presented as the mean ± standard deviation of three separate cultivations (*n* = 3 each)

**Fig. 7:**
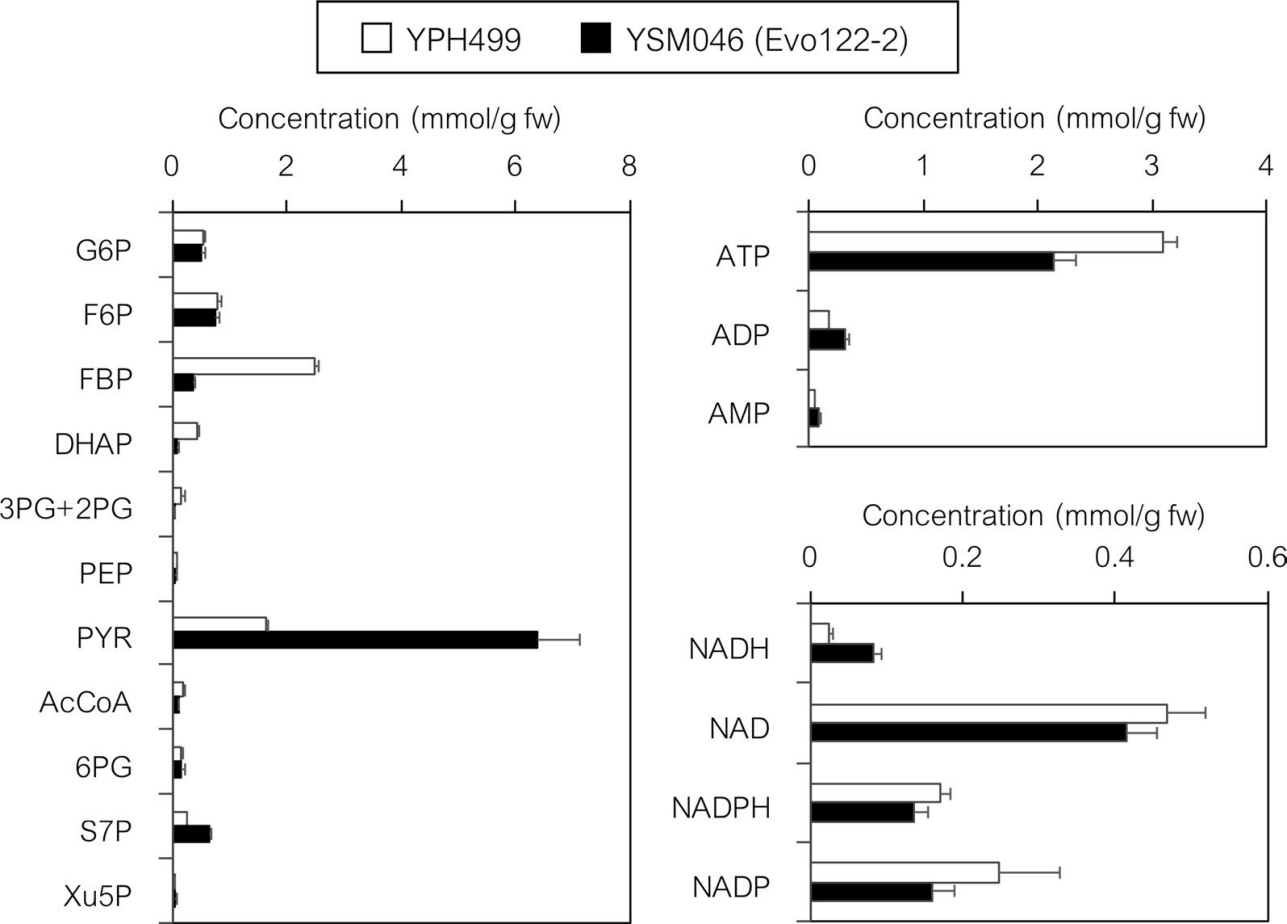
Metabolic profiling of the YPH499 and YSM046 (evolved PDCΔ) strains. Cells were cultured in a shake flask containing 100 mL of SD medium (20 g L^−1^ glucose) under aerobic conditions (the same cultures that provided the data shown in Fig. 6). Intracellular metabolites were extracted from cells collected at mid-log phase (OD_660_ = 1.0) and their concentrations were determined by ion-pairing LC–MS/MS analysis. G6P, glucose 6-phosphate; F6P, fructose 6-phosphate; FBP, fructose 1,6-bisphosphate; DHAP, dihydroxyacetone phosphate; 3PG, 3-phosphoglycerate; 2PG, 2-phosphoglycerate; PEP, phosphoenolpyruvate; PYR, pyruvate; AcCoA, acetyl-CoA; 6PG, 6-phosphogluconate; S7P, sedoheptulose 7-phosphate; Xu5P, xylulose 5-phosphate. Data are presented as the mean ± standard deviation of three separate cultivations (*n* = 3 each)

The state of central carbon metabolism in the evolved strain was investigated by metabolic profiling analysis. The wild-type strain (YPH499) and the evolved strain (YSM046, PDCΔ + evolved) (Table 1) were cultivated in shake flasks with 100 mL SD medium under aerobic conditions (Fig. 6). Glucose was slowly consumed by the YSM046 strain and no ethanol and little glycerol were produced (Fig. 6), consistent with the small-scale cultivation data (Additional file 1: Fig. S3). The specific growth rates of YPH499 and the evolved strain YSM046 were 0.30 ± 0.003 and 0.091 ± 0.002 h^−1^, respectively. Intracellular metabolites were extracted from cells collected at mid-log phase (OD_660_ = 1.0; see “Methods” for the detailed procedure) and metabolite concentrations were determined by ion-pairing LC–MS/MS analysis (Fig. 7).

Comparison of the pool sizes of glycolytic intermediates and cofactors indicated that two substrates for the PDC (pyruvate decarboxylase) and ADH (alcohol dehydrogenase) reactions, pyruvate and NADH, significantly accumulated (3.9- and 3.2-fold, respectively) in the evolved PDCΔ strain YSM046 cells (Fig. 7). The reduced levels of energy charge (decreased ATP and increased ADP) and fructose 1,6-bisphosphate (FBP: only 15.3% that of YPH499) in YSM046 cells suggested that the low ATP level should have affected the activity of phosphofructokinase (fructose 6-phosphate (F6P) + ATP → FBP + ADP), resulting in slower glycolysis in the YSM046 strain. However, the level of pyruvate accumulation was suitable for demonstrating our strategy and thus we further exploited the YSM046 strain to validate the utility of tugging the pyruvate flux for 2,3-BDO production.

### Construction of 2,3-BDO-producing evolved PDCΔ strains harboring high-activity ALS and downstream *aldcLlOp* and *BDH1* genes (YHI030 strain)

**Fig. 8:**
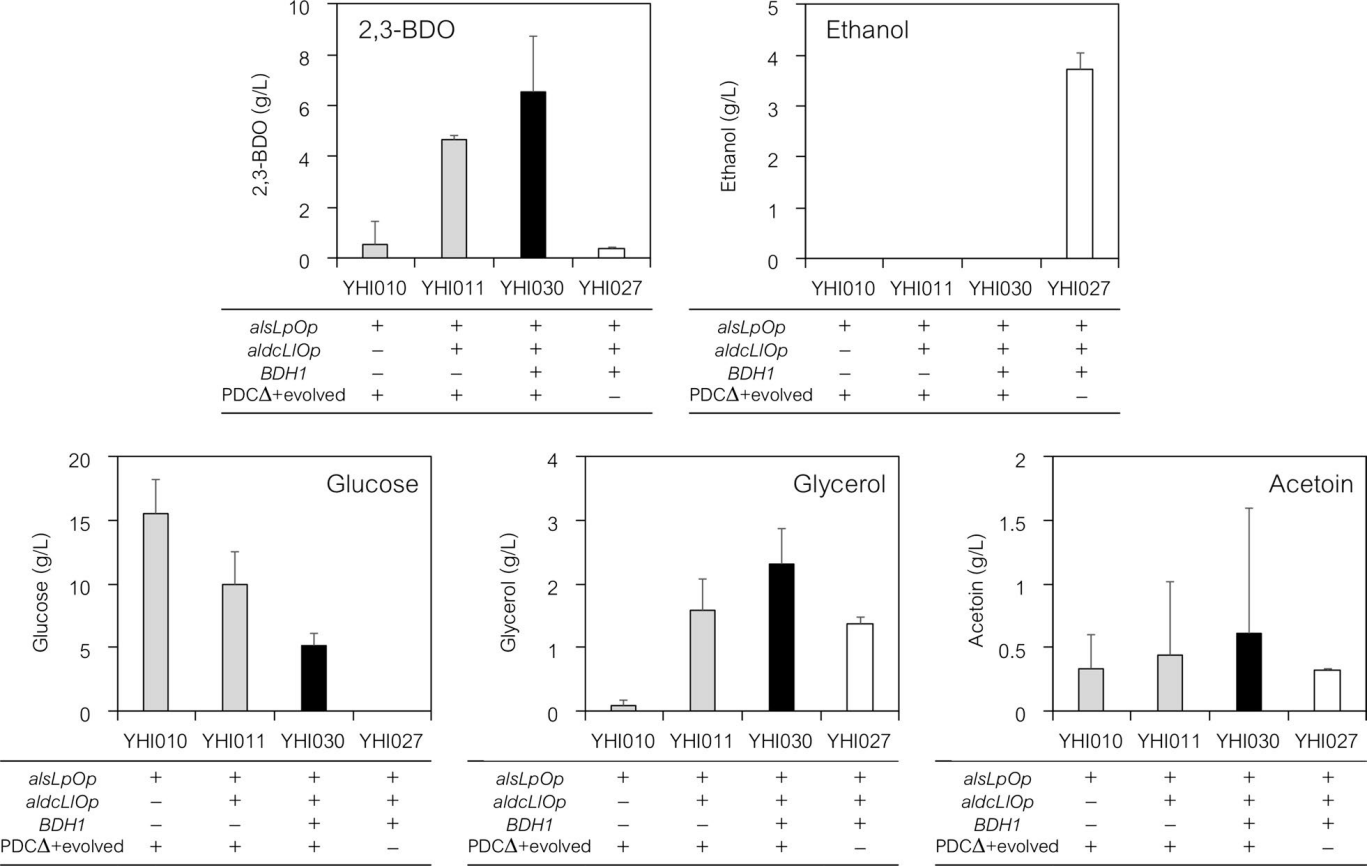
Performance of the YSM046 (evolved PDCΔ) strain expressing genes in the 2,3-BDO biosynthetic pathway. Lp, *Lactobacillus plantarum*; Ll, *Lactococcus lactis*. Op, codon optimized. One or two genes encoding high-activity ALS (*alsLpOp*) and/or ALDC (*aldcLlOp*) were expressed in the YSM046 strain (YHI010 and YHI011; gray bars). Three genes encoding high-activity ALS (*alsLpOp*), ALDC (*aldcLlOp*) and BDH (*BDH1* from *S. cerevisiae*) were also expressed in the YSM046 strain (YHI030; black bars). As a comparative control, the three genes (*alsLpOp*, *aldcLlOp* and *BDH1*) were expressed in the YPH499 wild-type strain (YHI027; white bars). The pATP422 and pAT425 vectors were used for expressing the ALS (*alsLpOp*), ALDC (*aldcLlOp*) and BDH (*BDH1*) genes. Fermentation experiments were performed in test tubes containing 5 mL of SD selection medium (20 g L^−1^ glucose) under semi-aerobic conditions. Metabolite concentrations in the medium were determined at 96 h after the start of fermentation. Data are presented as the mean ± standard deviation of three independent transformants (*n* = 3 each)

*Saccharomyces cerevisiae* strains producing 2,3-BDO were constructed by introducing the high-activity ALS (*alsLpOp*) gene and/or the downstream (ALDC and BDH; *aldcLlOp* and *BDH1*) genes for 2,3-BDO biosynthesis into the YSM046-evolved PDCΔ strain (YHI010, YHI011 and YHI030; Table 3) and the YPH499 wild-type strain (YHI027; Table 3). The strains were cultured in 5 mL of SD selection medium under semi-aerobic conditions. The concentrations of 2,3-BDO and other compounds 96 h after the start of fermentation are shown in Fig. 8.

Both glucose consumption and 2,3-BDO titer were increased by sequential introduction of the *alsLpOp*, *aldcLlOp* and *BDH1* genes into the YSM046 strains (Fig. 8). The 2,3-BDO titer of YHI030 strain (YSM046 strain expressing *alsLpOp*, *aldcLlOp* and *BDH1*) was 6.6 ± 2.2 g L^−1^ (0.41 g g^−1^ glucose consumed), comparable to the highest value reported for *S. cerevisiae* [12, 26]). We observed no over-production of 2,3-BDO by YHI027 strain expressing the same (*alsLpOp*, *aldcLlOp* and *BDH1*) genes present in wild-type YPH499 (0.37 ± 0.05 g L^−1^; 0.02 g g^−1^ glucose consumed). No ethanol was detected in the culture media of the YSM046-derived strains (YHI030, YHI011 and YHI010) whereas YHI027 strain produced 3.7 ± 0.3 g L^−1^ ethanol as the major product. YHI030 showed slightly higher (1.7-fold) glycerol production than YHI027 (Fig. 8), probably due to an excess of NADH resulting from the loss of ethanol biosynthesis. The higher growth rate of the evolved PDCΔ strain (YSM046) compared to the non-evolved PDCΔ strain (YSM021) was confirmed, even when YSM046 expressed the *alsLpOp*, *aldcLlOp* and *BDH1* genes (Table 3, Additional file 1: Fig. S5). These results indicate that the YSM046-evolved PDCΔ strain is useful as a host strain for tugging pyruvate carbon flux and increasing 2,3-BDO production, although its growth rate requires improvement prior to industrial application.

### 2,3-BDO production by fermentation using YHI030 strain

2,3-BDO was further produced by fermentation using the YHI030 strain. Small fermentation bottles containing SD selection medium and several high concentrations of glucose (25, 50 and 100 g L^−1^) as the sole carbon source were used to generate oxygen-limited (anaerobic) conditions.

**Fig. 9:**
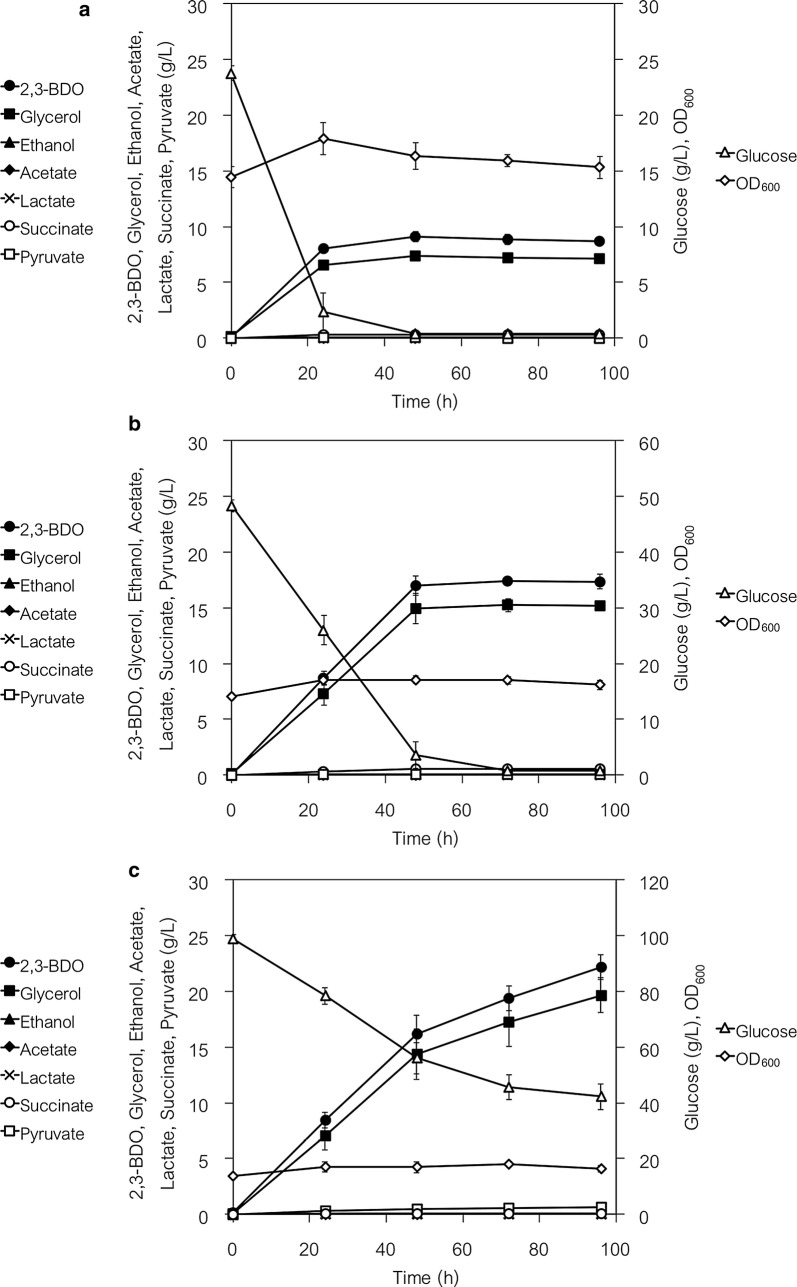
2,3-BDO fermentation by the YHI030 (evolved PDCΔ YSM046 expressing *alsLpOp*, *aldcLlOp* and *BDH1*) strain. After pre-cultivation, 0.75 g-wet weight of cells were transferred into 50 mL of fresh SD selection media containing 25 (**a**), 50 (**b**) or 100 (**c**) g L^−1^ glucose to give initial cell densities of 15 g-wet cell weight L^−1^. Fermentation was conducted in small fermentation bottles with CO_2_ gas outlets under anaerobic (oxygen-limited) conditions. Data are presented as the mean ± standard deviation of three separate cultivations (*n* = 3 each)

The cells almost completely consumed 25 and 50 g L^−1^ glucose after 24 and 48 h of fermentation, respectively (Fig. 9a, b). After 48 and 72 h of fermentation, the 2,3-BDO titer of YHI030 strain was 9.1 ± 0.4 g L^−1^ (0.37 g g^−1^ glucose consumed) and 17.4 ± 0.3 g L^−1^ (0.35 g g^−1^ glucose consumed), respectively, from 25 and 50 g L^−1^ glucose, and the glycerol titer was, respectively, 7.4 ± 0.2 g L^−1^ (0.30 g g^−1^ glucose consumed) and 15.3 ± 0.6 g L^−1^ (0.31 g g^−1^ glucose consumed). Although the cells produced 22.1 ± 1.1 g L^−1^ 2,3-BDO (0.38 g g^−1^ glucose consumed) and 19.7 ± 1.6 g L^−1^ glycerol (0.34 g g^−1^ glucose consumed) from 100 g L^−1^ glucose after 96 h of fermentation, the glucose consumption rate decreased after 48 h and the 100 g L^−1^ of glucose was not completely consumed (Fig. 9c). For all concentrations of glucose, the yields of 2,3-BDO and glycerol were, respectively, 0.35–0.38 and 0.30–0.34 g g^−1^ glucose consumed, whereas very low levels of the other metabolites measured (ethanol, acetate, lactate, succinate and pyruvate) were detected in the fermentation media. The relatively high levels of glycerol generation were probably caused by the excess of NADH arising from deficient ethanol biosynthesis (similar to the results shown in Fig. 8).

### Investigation of fermentation conditions for 2,3-BDO production by YHI030 strain

Since not all the 100 g L^−1^ glucose was consumed during fermentation (Fig. 9c), we investigated the effects of adding glucose in a step-by-step manner (Additional file 1: Fig. S6). During anaerobic fermentation, 50 or 25 g L^−1^ glucose was added to the culture in incremental steps (Additional file 1: Fig. S6A and B). In both cases, the cells showed decreased glucose consumption after the addition of a total of 50 g L^−1^ glucose. The 2,3-BDO titer remained static at around 20 g L^−1^.

Subsequently, we tested the effect of aerobic fermentation in SD selection medium (50 and 100 g L^−1^ glucose) using Erlenmeyer flasks and different agitation speeds (100 and 200 rpm) (Additional file 1: Fig. S7). At 100-rpm agitation, YHI030 strain produced approximately 20 g L^−1^ 2,3-BDO in medium containing initially either 50 or 100 g L^−1^ glucose (Additional file 1: Fig. S7A and B); in medium containing initially 100 g L^−1^ glucose, the cells consumed approximately 40 g L^−1^ glucose (Additional file 1: Fig. S7B). The production of glycerol was significantly reduced when fermentation was conducted in medium containing initially either 50 or 100 g L^−1^ glucose and agitated at 100 rpm (4.7 and 7.2 g L^−1^ glycerol, respectively) (Additional file 1: Fig. S7A and B).

The fermentation of either 50 or 100 g L^−1^ glucose under more aerobic conditions (200 rpm) resulted in lower 2,3-BDO production (~ 14 g L^−1^) and decreased glucose consumption (~ 30 g L^−1^) (Additional file 1: Fig. S7C and D), indicating that conditions approximating an oxygen-limited aerobic state are suitable for 2,3-BDO production by YHI030 strain. Furthermore, all cultures showed increased cell densities (OD = 20–25) and a significant decrease in pH (2–3) after 24 h of fermentation (Additional file 1: Fig. S7C and D). Fermentation at 300 rpm in medium containing 50 or 100 g L^−1^ glucose resulted in 2,3-BDO production profiles similar to those at obtained at 200 rpm (data not shown).

We investigated the effect of severely decreased pH on the production of 2,3-BDO by adding 200 mM MES buffer to the fermentation medium and adjusting the pH to 6.0, then conducting fermentation using milder agitation speeds (50, 100 and 150 rpm). We found that YHI030 strain rapidly and completely consumed 50 g L^−1^ glucose at each agitation speed tested (Additional file 1: Fig. S8). The cells produced approximately 14 g L^−1^ 2,3-BDO and 5 g L^−1^ glycerol during fermentation conducted at 100 and 150 rpm (Additional file 1: Fig. S8B and C), whereas at 50 rpm, the cells produced 17.6 g L^−1^ 2,3-BDO but no significant reduction in glycerol was observed (Additional file 1: Fig. S8A). The cell density was increased (OD = 25–28) and the pH was moderately decreased (pH = 3.5–4.5) during fermentation at 100 and 150 rpm but remained essentially unchanged (OD = 18–19, pH = 5.2–5.4) at 50 rpm.

YHI030 strain produced 34–39 g L^−1^ 2,3-BDO after 72–96 h of fermentation in pH-adjusted buffered medium containing 100 g L^−1^ glucose (Additional file 1: Fig. S9). At 50 rpm, the glucose was almost entirely consumed at 72 h (Additional file 1: Fig. S9A), whereas more than 10–15 g L^−1^ glucose remained after 96 h of fermentation at 100 and 150 rpm (Additional file 1: Fig. S9B and C). Similar to fermentation in the presence of 50 g L^−1^ glucose, the cell density and pH of cultures agitated at 50 rpm in the presence of 100 g L^−1^ glucose remained essentially constant whereas the cell densities of cultures agitated at 100 and 150 rpm were increased and the pH values of the media were decreased.

### Fed-batch fermentation by YHI030 strain for the production of 2,3-BDO by adding a high concentration of glucose

**Fig. 10:**
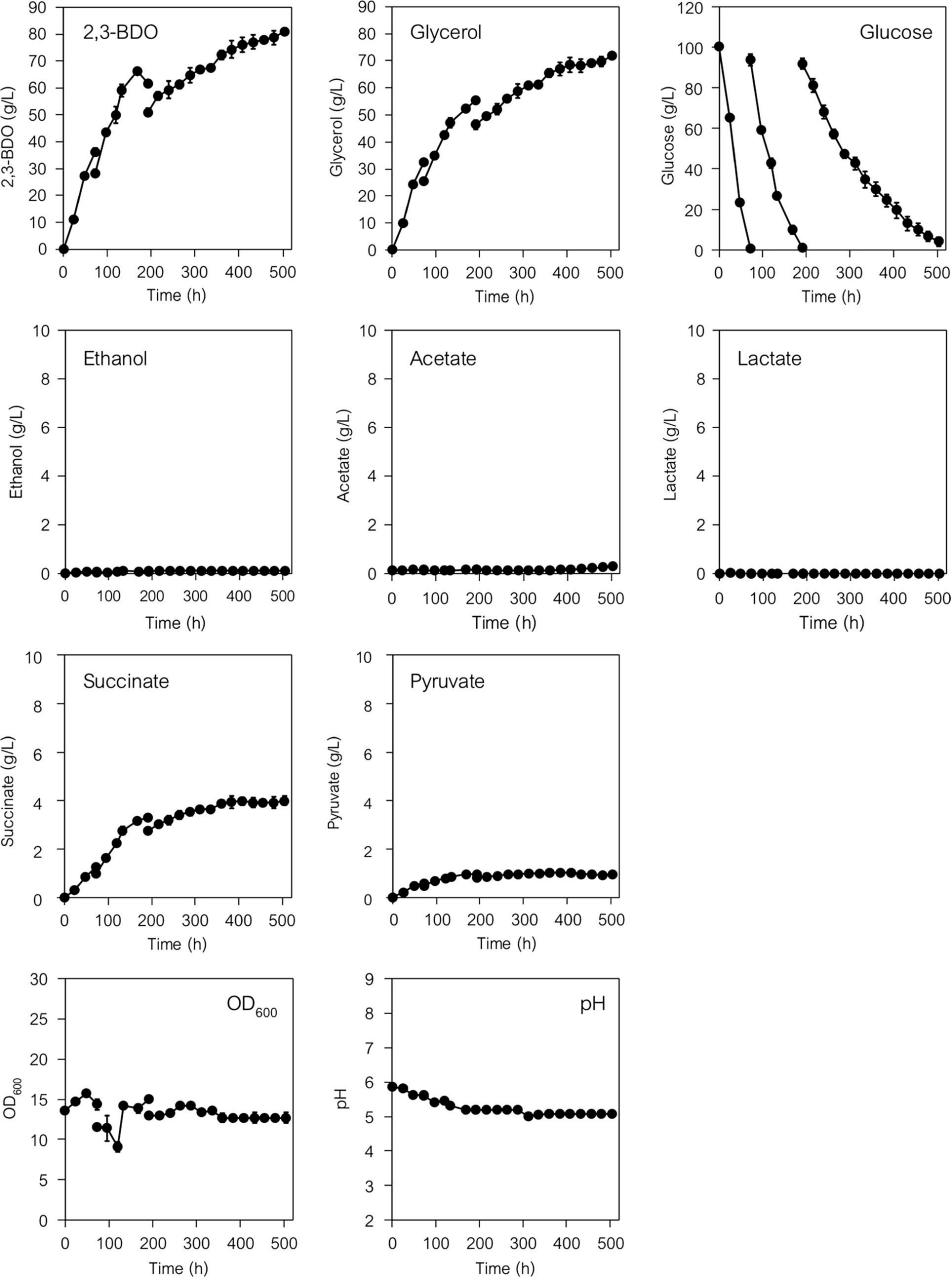
2,3-BDO fermentation by the YHI030 (evolved PDCΔ YSM046 expressing *alsLpOp*, *aldcLlOp* and *BDH1*) strain in pH-adjusted buffered medium under mild aerobic conditions. After pre-cultivation, 0.75 g-wet weight of cells were transferred into 50 mL of fresh SD selection medium containing 100 g L^−1^ glucose and 500 mM MES (pH adjusted to 6.0) to give initial cell densities of 15 g-wet cell weight L^−1^. Fermentation was conducted in Erlenmeyer flasks under mild aerobic conditions. The agitation speed was 50 rpm. A total of 300 g L^−1^ of glucose was added to the fermentation culture in three equal doses (100 g L^−1^ each, including the initially contained glucose). Glucose was added to the fermentation culture at 72 and 192 h. Data are presented as the mean ± standard deviation of three separate cultivations (*n* = 3 each)

Finally, we again tested the step-by-step addition of glucose as the sole carbon source to medium buffered with 500 mM MES (pH 6.0) (Fig. 10). During aerobic fermentation with 50-rpm agitation (mild aerobic conditions), 300 g L^−1^ glucose was added to the fermentation culture in three equal doses (100 g L^−1^ each). YHI030 strain consumed the initial 100 g L^−1^ glucose and produced 36.2 ± 1.7 g L^−1^ 2,3-BDO at 72 h (Fig. 10). The cells consumed an additional 100 g L^−1^ glucose (total 200 g L^−1^) and produced 61.7 ± 1.3 g L^−1^ 2,3-BDO at 120 h after the first glucose addition (total 192 h). Finally, the cells almost completely consumed a further additional 100 g L^−1^ glucose (total 300 g L^−1^) and the 2,3-BDO titer reached 81.0 ± 1.3 g L^−1^ at 312 h after the second glucose addition (total 504 h). The highest yield of 2,3-BDO in each (1st, 2nd and 3rd) fermentation step was 0.38 (at 72 h), 0.35 (at 96 h; total 168 h), and 0.27 (at 312 h; total 504 h) g g^−1^ glucose consumed. The glycerol titers were 32.3 ± 0.7 g L^−1^ (at 72 h), 55.3 ± 0.7 g L^−1^ (at 120 h; total 192 h) and 71.8 ± 0.7 g L^−1^ (at 312 h; total 504 h). The cells produced small amounts of succinate (1.3, 3.3 and 4.0 g L^−1^) and pyruvate (0.6, 1.0 and 1.0 g L^−1^) (at 72 h, at 120 h; total 192 h, and at 312 h; total 504 h), but ethanol, acetate or lactate were not detected. The cell density remained essentially unchanged during the fermentation and pH gradually decreased to between pH 5 and 6 (Fig. 10).

## Discussion

The aim of this study was to develop a yeast metabolic engineering strategy for increasing higher alcohol production and concomitantly reducing ethanol subgeneration. To this end, we proposed a methodological approach for tugging the carbon flux in central metabolism at an important hub branching point (e.g., pyruvate and acetyl-CoA). We verified the validity of this strategy by testing 2,3-BDO production, which requires pyruvate as the hub branching point.

First, we ensured that pyruvate flux would be tugged towards 2,3-BDO biosynthesis by searching various ALS (acetolactate synthase) candidates to serve as the common first-step enzyme for the conversion of pyruvate to 2,3-BDO. We identified a high-activity ALS gene from *L. plantarum* (*alsLp*) by analyzing crude yeast cell extracts using an enzyme activity assay (Fig. 2). The high-activity *alsLp* gene was codon-optimized (*alsLpOp*) and its feasibility for 2,3-BDO production was tested. Expression of the *alsLpOp* gene in YPH499 wild-type strain resulted in a higher titer of 2,3-BDO production compared to the expression of other ALS genes, including endogenous yeast *ILV2* (mitochondria) [4] and *ILV2c* (cytosol) [38] (Fig. 3a). Simple tuning of aeration and BDH expression resulted in a significant increase and decrease in 2,3-BDO production and ethanol subgeneration, respectively (Fig. 3b, c). Thus, we validated the strategy of pyruvate carbon flux tugging using high-activity ALS for increasing higher alcohol production (2,3-BDO) in the wild-type strain.

However, substantial ethanol production was still observed and thus we generated the evolved PDCΔ YSM046 strain by quadruple deletions (*MTH1*-Δ*T* allele and *PDC1, 5, 6*Δ) and laboratory evolution. LC–MS/MS analysis was used to compare the pool sizes of glycolytic intermediates and cofactors (Fig. 7) and showed that YSM046 is a promising strain for the production of 2,3-BDO and other compounds (e.g., lactate [44] and butanol [3]) because YSM046 significantly accumulated pyruvate and NADH, the precursor and cofactor required for the biosynthesis of various metabolites [23, 45]. However, the specific growth rate of evolved PDCΔ strain YSM046 was 0.091 ± 0.002 h^−1^ (from 20 g L^−1^ glucose under aerobic conditions), which is significantly slower than that of previously reported Pdc^−^ strains with similar *MTH1*-Δ*T* alleles (cultured in aerobic, pH-controlled bioreactors in medium containing 7.5 g L^−1^ glucose: 0.10 h^−1^ without evolution and 0.20 h^−1^ with evolution) [40, 46]. The same Pdc^−^ strains showed higher specific growth rates (0.24 and 0.23 h^−1^, respectively) following the addition of a small amount of ethanol (0.3%, v/v), whereas the original PDC-deficient yeast strain (without the *MTH1*-Δ*T* allele) could not grow in the presence of a high glucose concentration as the sole carbon source and required C2 compounds (ethanol or acetate) for growth under low glucose concentrations. The necessity for C2 compounds may be due to the inability to synthesize cytosolic acetyl-CoA from pyruvate [40, 46–48]. Thus, the addition of C2 compounds might improve both cell growth and 2,3-BDO production by PDCΔ strains at high glucose concentrations.

YSM046 (evolved PDCΔ) strain accumulated significant amounts of pyruvate (and NADH) and dramatically increased 2,3-BDO production by expressing *alsLpOp*, *aldcLlOp* and *BDH1* (Fig. 8). In contrast to YPH499 wild-type strain (Additional file 1: Fig. S2), the additional expression of ALDC (acetolactate decarboxylase; *aldcLlOp*) by YSM046 strain substantially improved 2,3-BDO productivity (Fig. 8). This evolved PDCΔ strain (YSM046) did not produce ethanol (Figs. 6 and 8) and thus the accumulated pyruvate (Fig. 7) could be used to consume glycolysis-derived NADH (or to regenerate NAD^+^). This excess pyruvate could be removed by converting 2-acetolactate to acetoin through spontaneous decarboxylation and subsequent reduction (via diacetyl) (Fig. 1b). This diacetyl pathway (Fig. 1b) is more balanced in terms of redox equilibrium than the ALDC pathway (Fig. 1c); however, the diacetyl pathway might have a limited capacity in 2-acetolactate conversion reaction. Consequently, expression of the ALDC enzyme likely aided the consumption of excess pyruvate during 2,3-BDO biosynthesis, despite the relatively poor redox balance (Fig. 1c).

The 2,3-BDO titer and yield of YHI030 strain (YSM046-evolved PDCΔ strain expressing the *alsLpOp*, *aldcLlOp* and *BDH1* genes) were 6.6 ± 2.2 g L^−1^ and 0.41 g g^−1^ consumed glucose, respectively. The 2,3-BDO titer generated by YHI030 strain was increased 17.8-fold compared to YHI027 strain (and 20.5-fold based on consumed glucose) by introducing the PDCΔ genotype and through evolutionary engineering. These data clearly demonstrate the utility of our strategy using pyruvate flux tugging for 2,3-BDO production. In addition, this yield is comparable to the highest yield reported for an engineered *S. cerevisiae* strain [12, 26]. In brief, the ADH- and NAD-dependent glycerol-3-phosphate dehydrogenase (GPD) enzymes were deleted from this previously reported strain (*adh1*–*5*Δ and *gpd1*–*2*Δ) and the *noxE* (NADH oxidase from *Lactococcus lactis*), *alsS* (ALS from *Bacillus subtilis*), *alsD* (ALDC from *B. subtilis*) and *BDH1* (*S. cerevisiae*) genes were inserted. This strain achieved a high yield of 2,3-BDO (72.9 g/L) at 0.41 g g^−1^ glucose consumed (fed-batch) by eliminating byproduct formation (GPD deletion) and redox rebalancing (*noxE* expression).

Finally, 2,3-BDO fermentation using a high density of YHI030 strain cells (15 g L^−1^; wet cell weight) and a high concentration of glucose as the sole carbon source was tested under various fermentation conditions (Figs. 9, 10, and Additional file 1: Figs. S6–S9). Under mild aerobic conditions (at an agitation of 50 rpm) in pH-adjusted buffered medium, YHI030 strain completely consumed both 50 and 100 g L^−1^ glucose and, respectively, produced 17.6 and 37.2 g L^−1^ 2,3-BDO (0.36 and 0.38 g g^−1^ glucose consumed) and glycerol (0.31 and 0.34 g g^−1^ glucose consumed) (Additional file 1: Figs. S8A and S9A). Fed-batch (consecutive addition) fermentation with 300 g L^−1^ glucose resulted in essentially complete consumption of the glucose and the production of 81.0 ± 1.3 g L^−1^ 2,3-BDO (at 504 h) (Fig. 10). Although 71.8 ± 0.7 g L^−1^ glycerol was produced as a major by-product, no ethanol was detected.

In the near future, the subgeneration of abundant glycerol will be decreased by more elaborate control of fermentation conditions than simple aeration and pH, and by genetic modifications such as expression of the *noxE* gene and deletion of the *GPD1* and *GPD2* genes, described previously [26, 49]. Other ethanol-reducing yeast strains (e.g., with ADH deletions [26, 50–53] and various PDC modifications [6, 49, 54]) can also be used to further demonstrate our pyruvate carbon flux tugging strategy, in addition to the PDCΔ strain. Kim et al. recently reported a 2,3-BDO production level of 154.3 g L^−1^ by *S. cerevisiae* (fed-batch fermentation) with fine-tuned *PDC* and *noxE* activities [54], and such strains might be compatible for further boosting 2,3-BDO production using our proposed strategy.

## Conclusions

In this study, we demonstrated that a strategy for tugging carbon flux can increase 2,3-BDO production and reduce ethanol subgeneration in Crabtree-positive *S. cerevisiae*. A stronger pulling effect at the hub branching point (tug of carbon flux) was critical in our strategy. Pyruvate was chosen as an example of an important hub compound because it links to both the TCA cycle and amino acid biosynthesis. A high-activity ALS and PDCΔ strain was effective in exerting the pyruvate pulling effect, achieving a high titer of 2,3-BDO and no ethanol subgeneration. This strategy could be used for the production of other chemicals such as isobutanol and 3-methyl-1-butanol (via valine biosynthesis) [55–57].

## Additional files

**Additional file 1.** Additional figures (Fig. S1–S9).

**Additional file 2.** Additional table (Table S1).

**Additional file 3.** Additional notes. This file includes Additional methods (Method S1–S9) and Additional results (Result S1 and S2).

## Abbreviations

2-MPA: 2-methylpropanal (isobutyraldehyde)
2,3-BDO: 2,3-butanediol
ADH: alcohol dehydrogenase
ALDC: acetolactate decarboxylase
ALS: acetolactate synthase
*alsLp*: ALS gene from *Lactobacillus plantarum*
*alsLpOp*: codon-optimized *alsLp* gene
*alsTf*: ALS gene from *Thermobifida fusca*
*alsTfOp*: codon-optimized *alsTf* gene
BDH: butanediol dehydrogenase
CDS: coding DNA sequence
F6P: fructose 6-phosphate
FBP: fructose 1,6-bisphosphate
*GPD*: glycerol-3-phosphate dehydrogenase gene
HPLC: high-performance liquid chromatography
IGV: Integrative Genomics Viewer
*ILV2c*: the truncated version of the *ILV2* gene lacking the mitochondrial signal sequence
*ilvBEc*: ALS gene from *Escherichia coli*
*ilvBEcOp*: codon-optimized *ilvBEc* gene
LC–MS/MS: liquid chromatography–tandem mass spectrometry
MEK: methyl ethyl ketone (butanone)
MES: 2-(*N*-morpholino)ethanesulfonic acid
*noxE*: NADH oxidase gene
OD: optical density
PDC: pyruvate decarboxylase
PDCΔ: PDC-deficient yeast
SD: synthetic dextrose
SC: synthetic complete
SNP: single nucleotide polymorphism
TCA: tricarboxylic acid
YPDA: yeast–peptone–dextrose–adenine
