# Supplementary material for: A pyruvate carbon flux tugging strategy for increasing 2,3-butanediol production and reducing ethanol subgeneration in the yeast Saccharomyces cerevisiae
Source: Biotechnol Biofuels. 2018 Jun 26;11:180. doi: 10.1186/s13068-018-1176-y (PMC6020211; doi:10.1186/s13068-018-1176-y)
Supplement: Supplementary file 1 — Additional file 1. Additional figures (Fig. S1–S9). [file 13068_2018_1176_MOESM1_ESM.pdf]

Biotechnology for Biofuels

Additional file 1:

Additional figures (Fig. S1–S9)

A pyruvate carbon flux tugging strategy for increasing 2,3-butanediol production and reducing ethanol subgeneration in the yeast *Saccharomyces cerevisiae*

Jun Ishii[a,*], Keisuke Morita[b], Kengo Ida[c], Hiroko Kato[a,b], Shohei Kinoshita[b], Shoko Hataya[a], Hiroshi Shimizu[b], Akihiko Kondo[a,c,d] and Fumio Matsuda[b,d,**]

[a] Graduate School of Science, Technology and Innovation, Kobe University, 1-1 Rokkodai, Nada, Kobe 657-8501, Japan

[b] Department of Bioinformatic Engineering, Graduate School of Information Science and Technology, Osaka University, 1-5 Yamadaoka, Suita, Osaka 565-0871, Japan

[c] Department of Chemical Science and Engineering, Graduate School of Engineering, Kobe University, 1-1 Rokkodai, Nada, Kobe 657-8501, Japan

[d] RIKEN Center for Sustainable Resource Science, 1-7-22 Suehiro, Tsurumi, Yokohama 230-0045, Japan

[**] **Corresponding author:** Fumio Matsuda

Department of Bioinformatic Engineering, Graduate School of Information Science and Technology, Osaka University, 1-5 Yamadaoka, Suita, Osaka 565-0871, Japan

Tel: +81-6-6879-7433, E-mail: fmatsuda@ist.osaka-u.ac.jp

[*] **Co-corresponding author:** Jun Ishii

Graduate School of Science, Technology and Innovation, Kobe University, 1-1 Rokkodai, Nada, Kobe 657-8501, Japan

Tel: +81-78-803-6356, E-mail: junjun@port.kobe-u.ac.jp

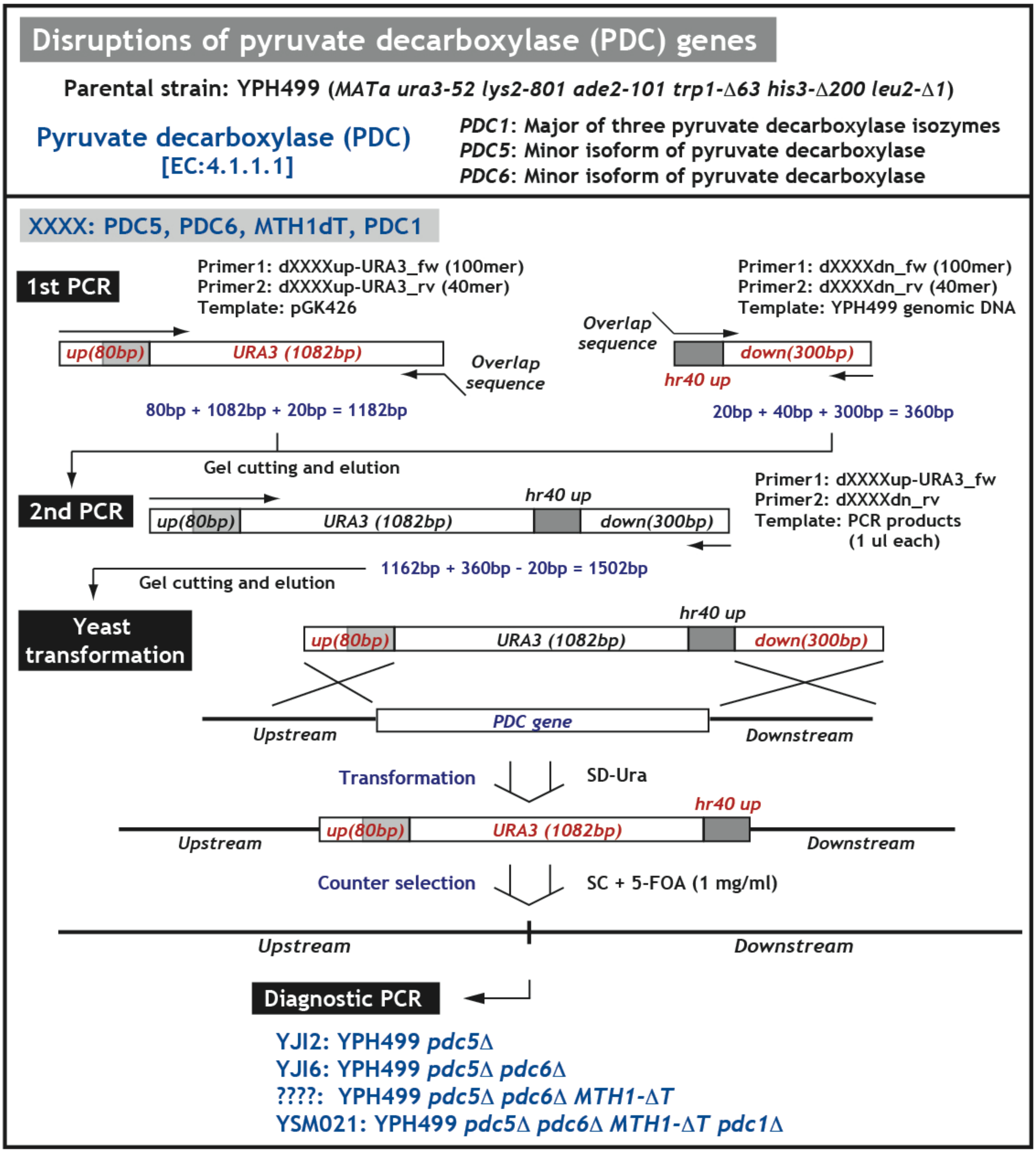

Fig. S1. **Flow diagram for construction of PDC-deficient yeast (PDCΔ) strain using the seamless marker recycling (URA-blaster) method.**

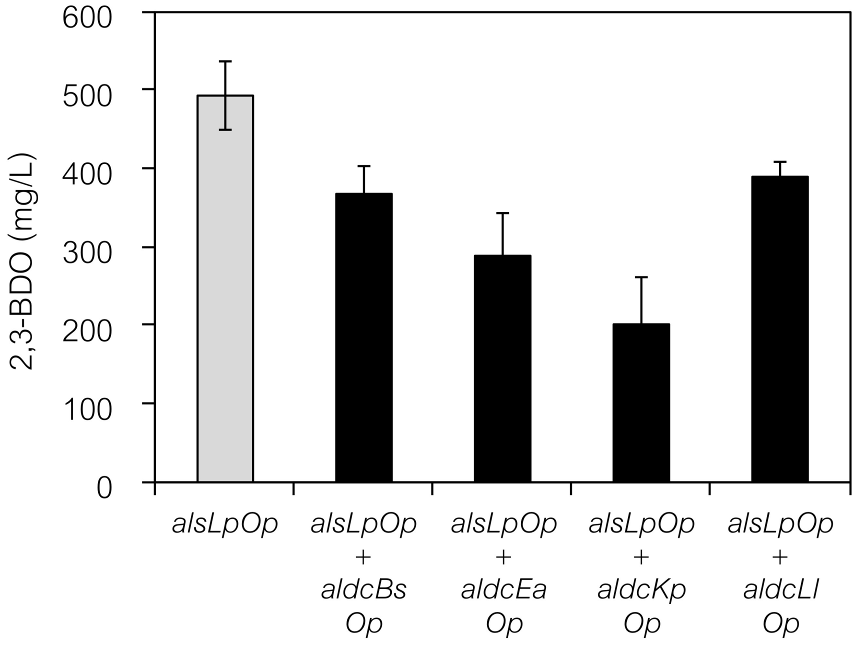

Fig. S2. 2,3-BDO productions of YPH499 strains co-expressing high-activity ALS (*alsLpOp* gene) and several ALDCs (*aldcOp* genes) (black bars; YIDB030–033). Lp, *Lactobacillus plantarum*; Bs, *Bacillus subtilis*; Ea, *Enterobacter aerogenes*; Kp, *Klebsiella pneumoniae*; Ll, *Lactococcus lactis*. YPH499 expressing only the ALS (*alsLpOp*) gene was used as a comparative control (gray bar). The pATP422 vector was used for expressing the ALS and ALDC genes. All ALS and ALDC genes were codon optimized using GeneArt GeneOptimizer software. Fermentations were performed in test tubes containing 3 mL of SD selection medium (20 g L^{-1} glucose) under semi-aerobic conditions. Concentrations of 2,3-BDO in the medium were determined at 48 h after the start of fermentation. Data are presented as the mean ± standard deviation of three independent transformants (n = 3 each).

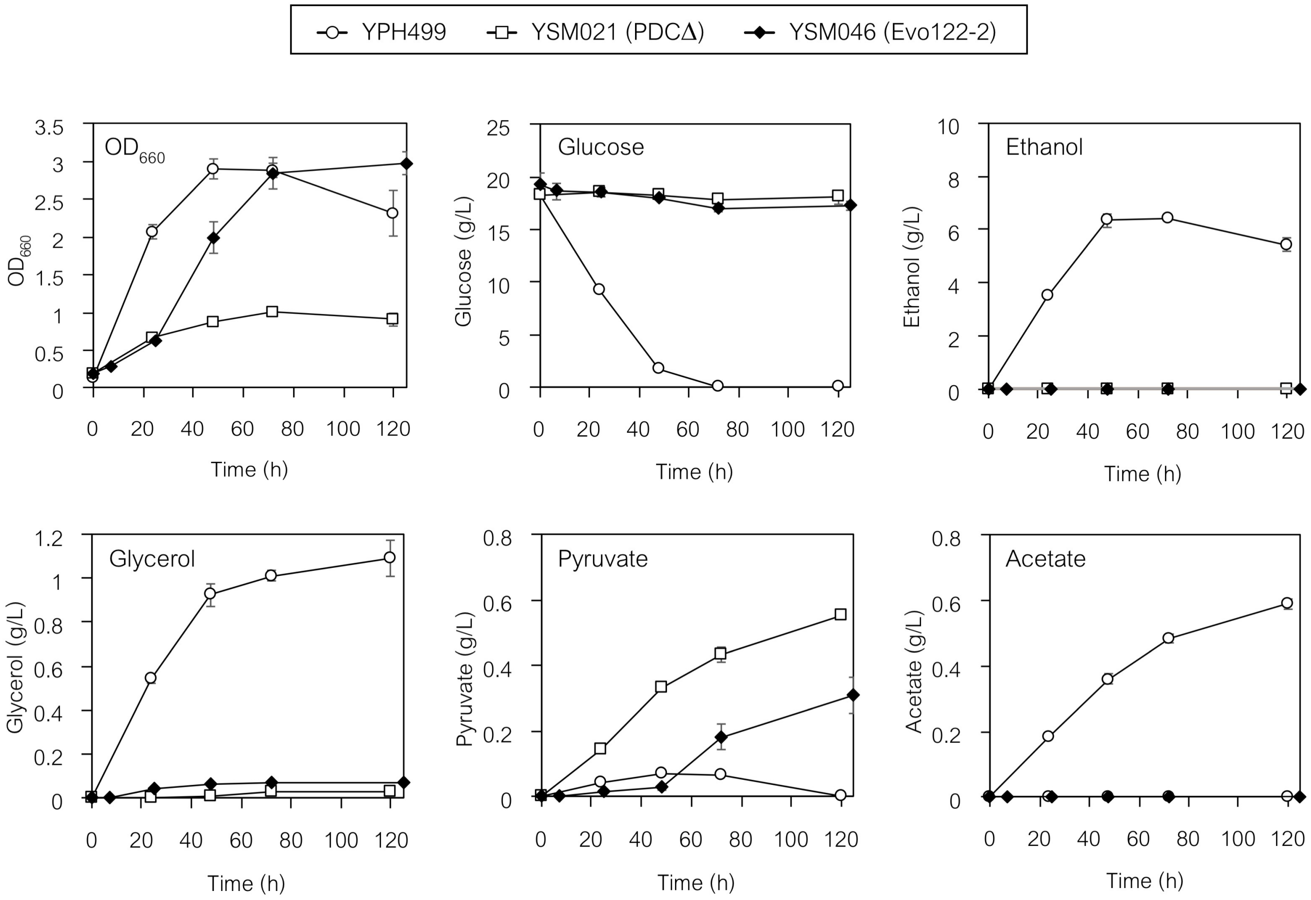

Fig. S3. Culture profiles of YPH499 (wild-type), PDCΔ (YSM021) and its evolved (YSM046; evolved PDCΔ) strains under semi-aerobic conditions. Cells were cultured in test tubes containing 5 mL of SD medium (20 g L^{-1} glucose). Data are presented as the mean ± standard deviation of three independent transformants (*n* = 3 each).

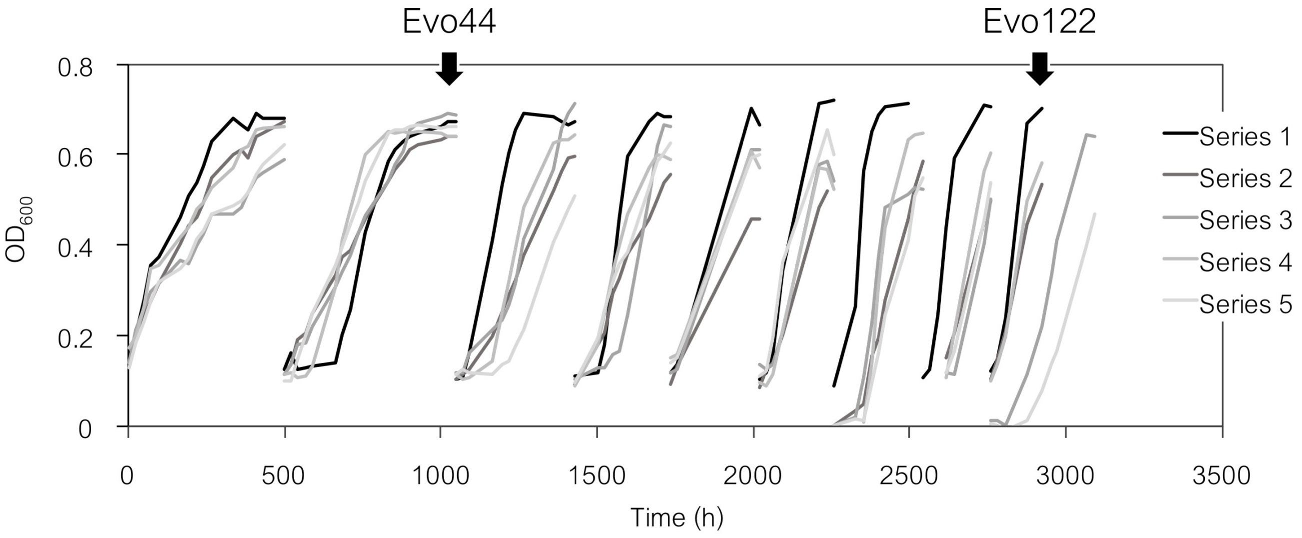

Fig. S4. Laboratory evolution of PDCΔ (YSM021) strains. The cells were cultivated in SD medium with five independent culture series. They were repeatedly transferred to the new medium when the cell density reached approximately OD_{600} = 0.7. The experiment was terminated at around 3,000 h, since further improvement was not observed.

	Host	*alsLp Op*	*aldcLl Op*	*BDH1*	*PDC*Δ	Evolution
-○- YIDB035	YPH499	+	+	+	–	–
-□- YSHB001	YSM021	+	+	+	+	–
-◆- YHI030	YSM046	+	+	+	+	+

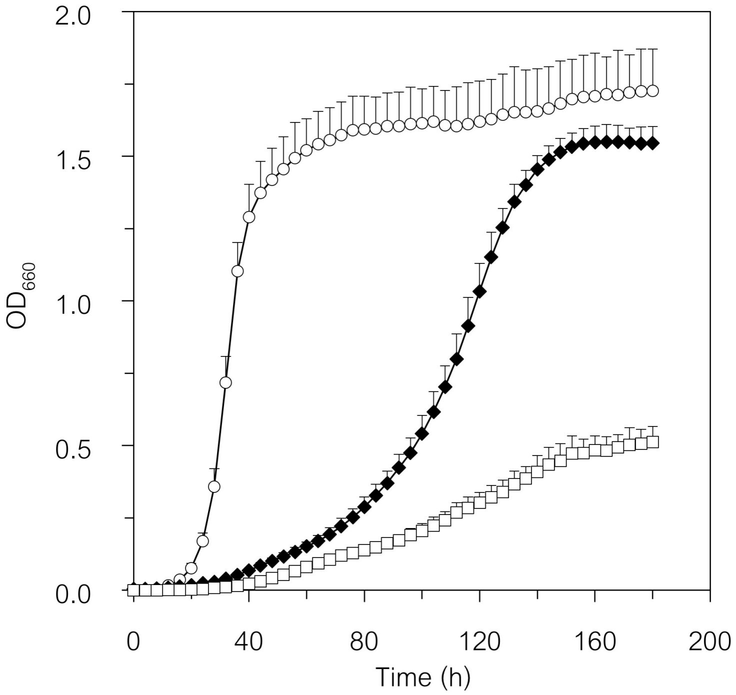

Fig. S5. Growth of YPH499 (wild-type), PDCΔ (YSM021) and its evolved (YSM046; evolved PDCΔ) strains expressing genes of 2,3-BDO biosynthetic pathway. YIDB035, YSHB001 and YHI030 respectively indicate the YPH499, YSM021 and YSM046 strains harboring pATP422-alsLpOp-aldcLlOp and pAT425-BDH1. Cells were cultured in 5 mL of SD selection medium (20 g L^{-1} glucose), and cell growth was automatically monitored every 4 h using a TVS062CA biophotorecorder. Data are presented as the mean ± standard deviation of three independent transformants (*n* = 3 each).

A

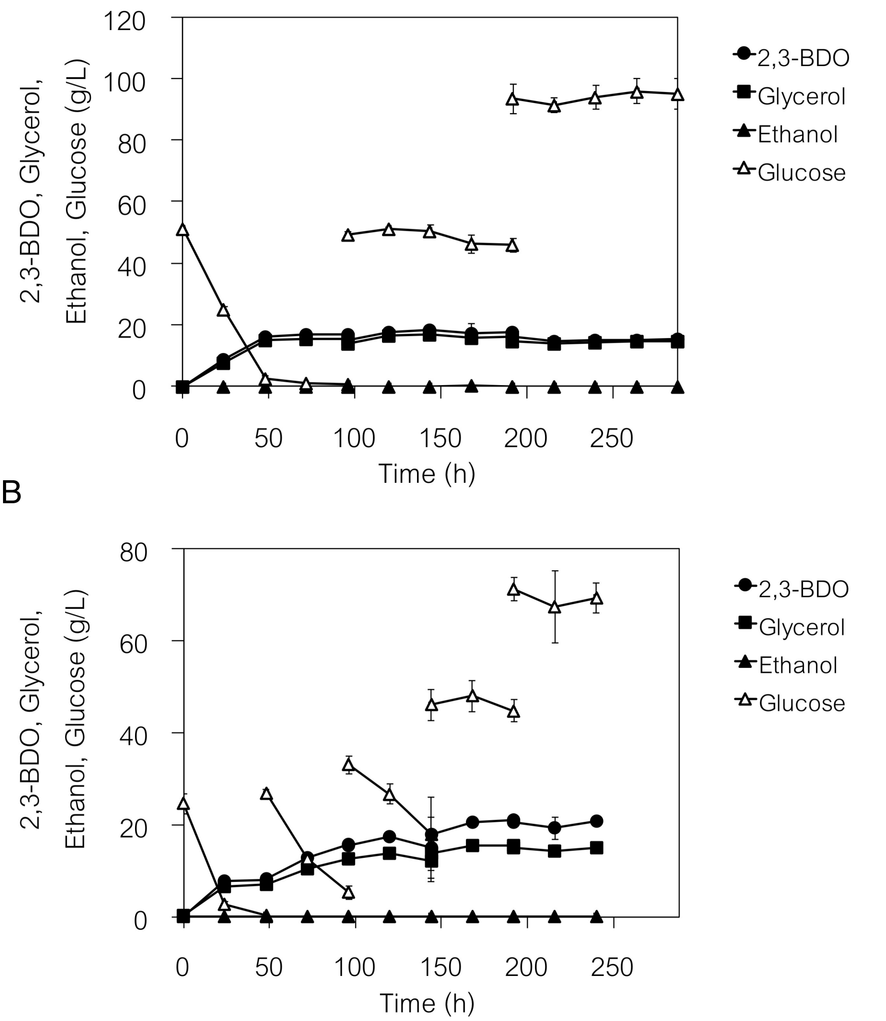

Fig. S6. Step-by-step addition of glucose for 2,3-BDO fermentations using YHI030 strain. After pre-cultivation, 0.75 g-wet weight of cells were respectively transferred into 50 mL of fresh SD selection media containing different concentrations of glucose (50 and 25 g L^{-1}) to give initial cell densities of 15 g-wet cell weight L^{-1}. Fermentations were started in small fermentation bottles with CO_2 gas outlets under anaerobic (oxygen-limited) conditions. (A) The SD selection medium initially contained 50 g L^{-1} of glucose, and additional glucose was consecutively added two times (at 96 and 192 h) to give each a concentration of 50 g L^{-1}. (B) The SD selection medium initially contained 25 g L^{-1} of glucose, and additional glucose was consecutively added four times (at 48, 96, 144 and 192 h) to give each a concentration of 25 g L^{-1}. Data are presented as the mean ± standard deviation of three separate cultivations (n = 3 each).

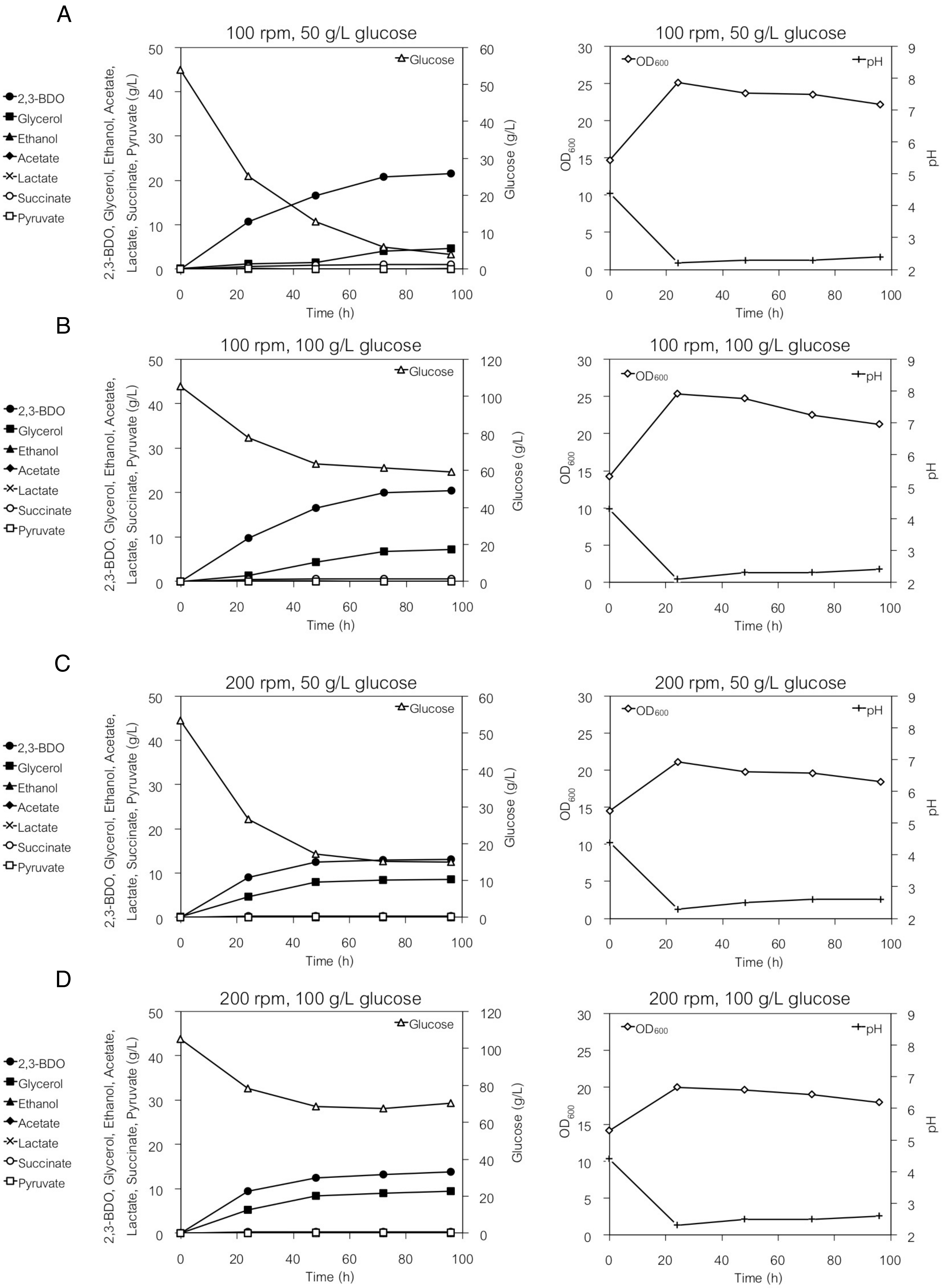
A
100 rpm, 50 g/L glucose
2,3-BDO
Glycerol
Ethanol
Acetate
Lactate
Succinate
Pyruvate
Glucose
2,3-BDO, Glycerol, Ethanol, Acetate, Lactate, Succinate, Pyruvate (g/L)
Glucose (g/L)
Time (h)
OD600
pH
B
100 rpm, 100 g/L glucose
C
200 rpm, 50 g/L glucose
D
200 rpm, 100 g/L glucose

Fig. S7. Consideration of aeration conditions (different agitation speeds) for 2,3-BDO fermentations using YHI030 strain in SD selection media containing 50 and 100 g L^{-1} glucose. After pre-cultivation, 0.75 g-wet weight of cells were respectively transferred into 50 mL of fresh SD selection media containing 50 g L^{-1} (A and C) and 100 g L^{-1} (B and D) glucose to give initial cell densities of 15 g-wet cell weight L^{-1}. Fermentations were started in Erlenmeyer flasks under aerobic conditions. Agitation speeds were set at 100 rpm (A and B) and 200 rpm (C and D).

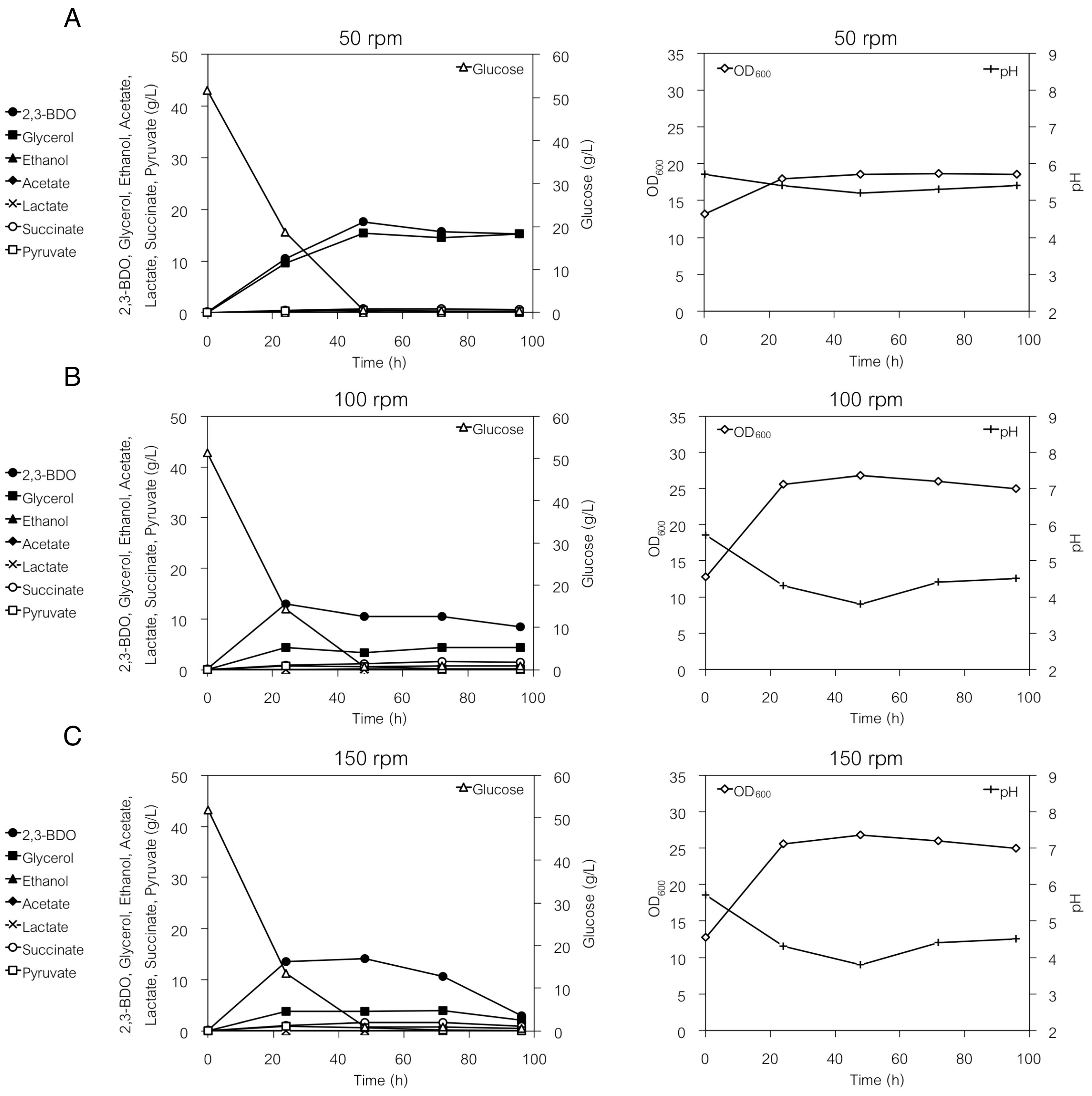

Fig. S8. Consideration of pH and aeration conditions for 2,3-BDO fermentations using YHI030 strain in buffered media containing 50 g L^{-1} glucose. After pre-cultivation, 0.75 g-wet weight of cells were respectively transferred into 50 mL of fresh SD selection media (50 g L^{-1} glucose) containing 200 mM MES (pH adjusted to 6.0) to give initial cell densities of 15 g-wet cell weight L^{-1}. Fermentations were started in Erlenmeyer flasks under mild aerobic conditions. Agitation speeds were set at 50 (A), 100 (B) and 150 (C) rpm.

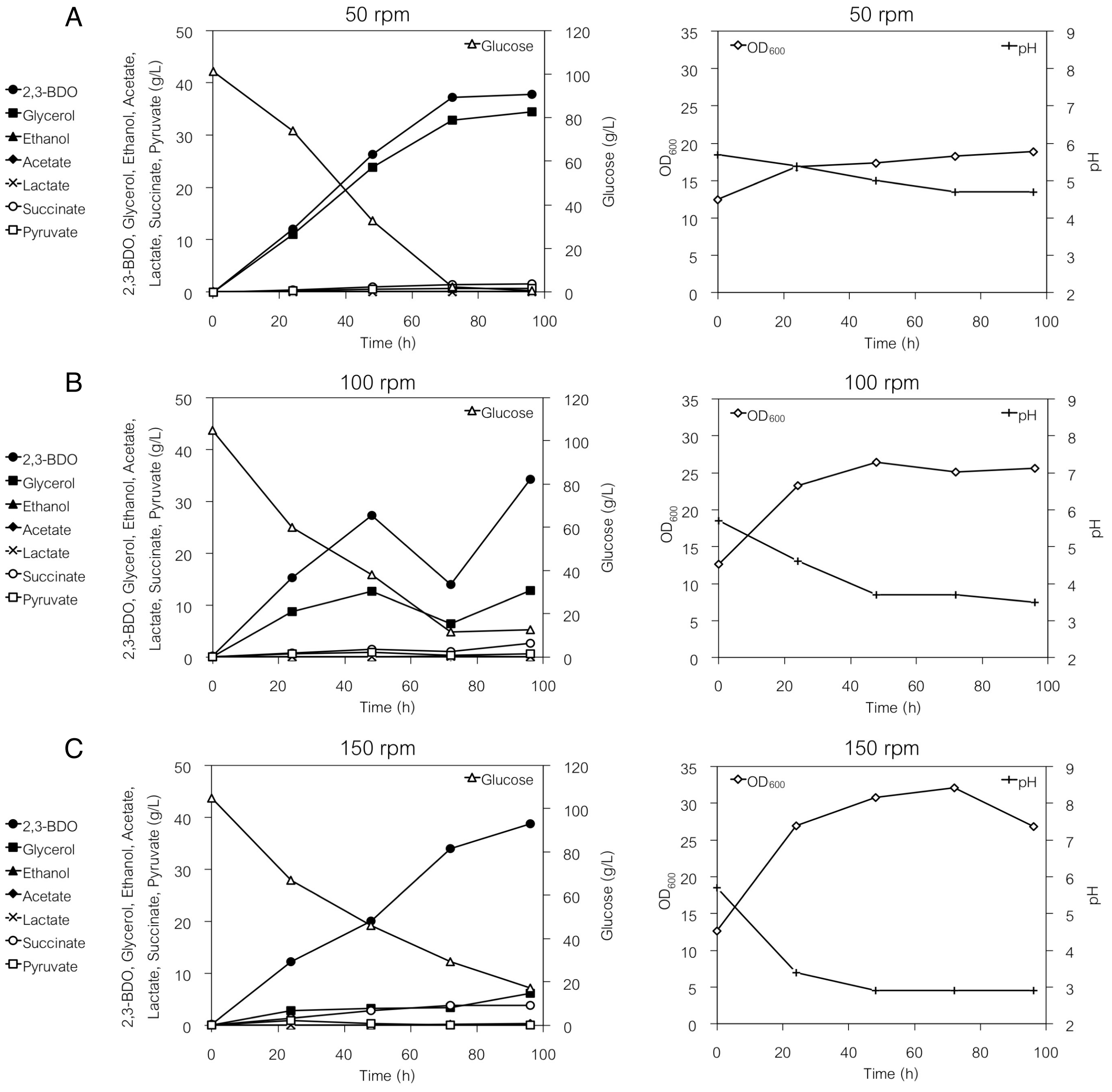

Fig. S9. Consideration of pH and aeration conditions for 2,3-BDO fermentations using YHI030 strain in buffered media containing 100 g L^{-1} glucose. After pre-cultivation, 0.75 g-wet weight of cells were respectively transferred into 50 mL of fresh SD selection media (100 g L^{-1} glucose) containing 200 mM MES (pH adjusted to 6.0) to give initial cell densities of 15 g-wet cell weight L^{-1}. Fermentations were started in Erlenmeyer flasks under mild aerobic conditions. Agitation speeds were set at 50 (A), 100 (B) and 150 (C) rpm.
