# Supplementary material for: A pyruvate carbon flux tugging strategy for increasing 2,3-butanediol production and reducing ethanol subgeneration in the yeast Saccharomyces cerevisiae
Source: Biotechnol Biofuels. 2018 Jun 26;11:180. doi: 10.1186/s13068-018-1176-y (PMC6020211; doi:10.1186/s13068-018-1176-y)
Supplement: Supplementary file 2 — Additional file 2. Additional table (Table S1). [file 13068_2018_1176_MOESM2_ESM.pdf]

Biotechnology for Biofuels
Additional file 2:

Additional table (Table S1)

A pyruvate carbon flux tugging strategy for increasing 2,3-butanediol production and reducing ethanol subgeneration in the yeast *Saccharomyces cerevisiae*

Jun Ishii[a,*], Keisuke Morita[b], Kengo Ida[c], Hiroko Kato[a,b], Shohei Kinoshita[b], Shoko Hataya[a], Hiroshi Shimizu[b], Akihiko Kondo[a,c,d] and Fumio Matsuda[b,d,**]

[a] Graduate School of Science, Technology and Innovation, Kobe University, 1-1 Rokkodai, Nada, Kobe 657-8501, Japan

[b] Department of Bioinformatic Engineering, Graduate School of Information Science and Technology, Osaka University, 1-5 Yamadaoka, Suita, Osaka 565-0871, Japan

[c] Department of Chemical Science and Engineering, Graduate School of Engineering, Kobe University, 1-1 Rokkodai, Nada, Kobe 657-8501, Japan

[d] RIKEN Center for Sustainable Resource Science, 1-7-22 Suehiro, Tsurumi, Yokohama 230-0045, Japan

[**] **Corresponding author:** Fumio Matsuda
Department of Bioinformatic Engineering, Graduate School of Information Science and Technology, Osaka University, 1-5 Yamadaoka, Suita, Osaka 565-0871, Japan
Tel: +81-6-6879-7433, E-mail: fmatsuda@ist.osaka-u.ac.jp

[*] **Co-corresponding author:** Jun Ishii
Graduate School of Science, Technology and Innovation, Kobe University, 1-1 Rokkodai, Nada, Kobe 657-8501, Japan
Tel: +81-78-803-6356, E-mail: junjun@port.kobe-u.ac.jp

Table S1
List of primers.

No.	Primer	Sequence
1	PDC5_URAb_1<br>(dPDC5up-URA3_fw)	5′- TTTCCTCTCTTTCTTATTACACTTATTTCACATAATCAATCTCAAAGAGAACAACA<br>CAATACAATAACAAGAAGAACAAATTTTTTGTTCTTTTTTTTGA
2	PDC5_URAb_2<br>(dPDC5up-URA3_rv)	5′- ATTGTGTTGTTCTCTTTGAGGGGTAATAACTGATATAATT
3	PDC5_URAb_3<br>(dPDC5dn_fw)	5′- AATTATATCAGTTATTACCCCTCAAAGAGAACAACACAATACAATAACAAGAAGA<br>ACAAAGCTAATTAACATAAAACTCATGATTCAACGTTTGTGTATT
4	PDC5_URAb_4<br>(dPDC5dn_rv)	5′- CTTCAATTAGCTAAGATCATAGCTAAAGGTACAAAACCGA
5	PDC5_URAb_check_fw	5′- TGCATACTTTATGCGTTTATGCGTTTTGCGCCCCTTGGAA
6	PDC5_URAb_check_rv	5′- AAAAAAAGCTTGTGAATAAAAATCTTTCGCTAAAAATCAA
7	PDC6_URAb_1<br>(dPDC6up-URA3_fw)	5′- CTTGTGCTATTGCAGTCCTCTTTTATATACAGTATAAATAAAAAACCCACGTAATAT<br>AGCAAAAACATATTGCCAACAAATTTTTTGTTCTTTTTTTTGA
8	PDC6_URAb_2<br>(dPDC6up-URA3_rv)	5′- GCTATATTACGTGGGTTTTTGGGTAATAACTGATATAATT
9	PDC6_URAb_3<br>(dPDC6dn_fw)	5′- AATTATATCAGTTATTACCCAAAAACCCACGTAATATAGCAAAAACATATTGCCAA<br>CAAAGCCATTAGTAGTGTACTCAAACGAATTATTGTTGCAAATA
10	PDC6_URAb_4<br>(dPDC6dn_rv)	5′- TTTATAAGACAAGCGCAGGGCCAAAGAAAAAATAATAATT
11	PDC6_URAb_check_fw	5′- CCCACACCAAAGGATGTTTTTTTATATCACGTTCCCTTTT

12	PDC6_URAb_check_rv	5′- GAGATGAGCCAAAGCAGAAAAACAGGGGACGCCGCCCTTC
13	MTH1dT_URAb_1<br>(MTH1dTup-URA3_fw)	5′- CAAGCTCCCTACAGGCCATTCCGGAAAACACGATGAGTGGCAGTGATAATGCTT<br>CTTTTCAAAGTTTGCCACTATCAATGTTTTTTGTTCTTTTTTTTGA
14	MTH1dT_URAb_2<br>(MTH1dTup-URA3_rv)	5′- GAAAAGAAGCATTATCACTGGGGTAATAACTGATATAATT
15	MTH1dT_URAb_3<br>(MTH1dTdn_fw)	5′- AATTATATCAGTTATTACCCCAGTGATAATGCTTCTTTTCAAAGTTTGCCACTATCA<br>ATGTTTTCTGCCCCCTCTACTGTGCACACGCAACTAACTAATG
16	MTH1dT_URAb_4<br>(MTH1dTdn_rv)	5′- ACCCACCGTTATTACTTCCGGTAGTTGATTACCCCATTCG
17	MTH1dT_URAb_check_f<br>w	5′- CAAATGACATTTTTCCTTTCTTCTCAAACTTTGTAATGCG
18	MTH1dT_URAb_check_rv	5′- TAGCCTCCATGTACAGATCCGAATGGACTAACGTTGCGAT
19	PDC1_URAb_1<br>(dPDC1up-URA3_fw)	5′- TATTTCTTGTCATATTCCTTTCTCAATTATTATTTTCTACTCATAACCTCACGCAAAA<br>TAACACAGTCAAATCAATCAAATTTTTTGTTCTTTTTTTTGA
20	PDC1_URAb_2<br>(dPDC1up-URA3_rv)	5′- TATTTTGCGTGAGGTTATGAGGGTAATAACTGATATAATT
21	PDC1_URAb_3<br>(dPDC1dn_fw)	5′- AATTATATCAGTTATTACCCTCATAACCTCACGCAAAATAACACAGTCAAATCAAT<br>CAAAGCGATTTAATCTCTAATTATTAGTTAAAGTTTTATAAGCA
22	PDC1_URAb_4<br>(dPDC1dn_rv)	5′- TGTTCCTTAATCAAGGATACCTCTTTTTTTTTCCTTGGTT
23	PDC1_URAb_check_fw	5′- TGGAATAATCAATCAATTGAGGATTTTATGCAAATATCGT
24	PDC1_URAb_check_rv	5′- GGCAGTTTTGAATTGAGTAACCATTATTTGTATCGAGGTG
25	InF_XmaI_ALSAo_fw	5′- TAGTGGATCCCCCGGGATGGCCGATGAAAGTACTGTGGGAAGC

26	InF_XmaI_ALSAo_rv	5′- ATTCTCTAGACCCGGGCTACCTAATAACGATATCCAAGACAACTG
27	NheI_alsSBs_fw	5′- AAAAGCTAGCATGACAAAAGCAACAAAAGAACAAAAATCC
28	BglII_alsSBs_rv	5′- AAAAAGATCTCTAGAGAGCTTTCGTTTTCATGAGTTCCCC
29	NheI_alsCg_fw	5′- AATTGCTAGCGTGAATGTGGCAGCTTCTCAACAGCCCAC
30	XmaI_als1Cg_rv	5′- AATTCCCGGGTTATGCCTCGGTCGATTCAACGGCGGCATC
31	InF_SalI_ilvBEc_fw	5′- AAACGCTAGCGTCGACATGGCAAGTTCGGGCACAAC
32	InF_XmaI_ilvBEc_rv	5′- ATTCTCTAGACCCGGGTTATTCCCCCACCATTTCAG
33	InF_SalI_ilvGEc_fw	5′- AAACGCTAGCGTCGACATGAATGGCGCACAGTGGGT
34	InF_XmaI_ilvGEc_rv	5′- ATTCTCTAGACCCGGGTCATGATAATTTCTCCAACA
35	InF_SalI_ilvIEc_fw	5′- AAACGCTAGCGTCGACATGGAGATGTTGTCTGGAGC
36	Inf_XmaI_ilvIEc_rv	5′- ATTCTCTAGACCCGGGTCAGGTTCTCTCCGTTTTGC
37	InF_SalI_AHAS2Gm_fw	5′- AAACGCTAGCGTCGACATGGCGGCCACAGCTTCCAGAACCAC
38	InF_XmaI_AHAS2Gm_rv	5′- ATTCTCTAGACCCGGGTCAGTACCTCGTTCTACCGTCTCCCTC
39	SalI_AHAS3Gm_fw	5′- AATTGTCGACATGGCGGCTATAACTGCCCCAAAAGCTGCG
40	SalI_AHAS3Gm_fw	5′- AATTCCCGGGTCAGTAACTTGTCCTGCCATCACCCTCAG
41	InF_SalI_ilvGHe-1_fw	5′- AAACGCTAGCGTCGACATGCATGACACCGACCGACACGACATGACC
42	InF_XmaI_ilvGHe-1_rv	5′- ATTCTCTAGACCCGGGTCAGTCGTGACCGATGGCATCGGCCCA
43	InF_SalI_ilvGHe-2_fw	5′- AAACGCTAGCGTCGACATGACTTGTGCCCAGCGACTGATCCAGCTC
44	InF_XmaI_ilvGHe-2_rv	5′- ATTCTCTAGACCCGGGCTAAAGTGAGTCGGCCCAGCCGGCGGCGTC
45	SalI_alsLl_fw	5′- ATACGTCGACATGTCTGAGAAACAATTTGGGGCGAACTTGGTTGTCGATAGTTTG
46	XmaI_alsLl_rv	5′- CGTACCCGGGTCAATAAAACTCTTCAGGCAATAATTTTTCTGCTAATTTAATG
47	BamHI_alsLp_fw	5′- ATATGGATCCATGCCAGATAAGAAATATTACGGGGCCGAT
48	XmaI_alsLp_rv	5′- ATATCCCGGGTTAATAAAACTGGTCTGGTAACATTGTCTT

49	NheI_alsSg-1_fw	5′-	ATATGCTAGCGTGAGCCACGACCACGACGACCGGCCGCAG
50	BamHI_alsSg-1_rv	5′-	ATATGGATCCCTACAGGTGCGTCGGCTCGAACATCCGCAG
51	NheI_alsSg-2_fw	5′-	ATATGCTAGCATGATCAGCGTGAGCGACGCCGAGCACCGG
52	BamHI_alsSg-2_rv	5′-	ATATGGATCCTCAGAGCGCCGAGTCGAGGTGCCCGAGGTG
53	NheI_alsTf_fw	5′-	AATTGCTAGCATGACCGAGCAGATGACCGGAGCCCAATCG
54	XmaI_alsTf_rv	5′-	AATTCCCGGGCTATTCTTCCTCGTCGTCCCATTCAGGCGC
55	BamHI_ALS1Zm_fw	5′-	AATTGGATCCATGGAGATGGAGATCCACCAGGCACTCACC
56	XmaI_ALS1Zm_rv	5′-	AATTCCCGGGTCAGTACACAGTCCTGCCATCACCATCCAG
57	InF_SalI_alsLpOp_fw	5′-	AAACGCTAGCGTCGACATGCCAGACAAAAAGTATTATGGTGCCGAT
58	InF_XmaI_alsLpOp_rv	5′-	ATTCTCTAGACCCGGGTTAGTAGAATTGGTCTGGCAACATAGTTTT
59	InF_SalI_ilvBEcOp_fw	5′-	AAACGCTAGCGTCGACATGGCTTCTTCTGGTACTACTTCCACCAGA
60	InF_XmaI_ilvBEcOp_rv	5′-	ATTCTCTAGACCCGGGTCATTCACCAACCATTTCAGTATTTGCAGC
61	InF_SalI_alsTfOp_fw	5′-	AAACGCTAGCGTCGACATGACTGAACAAATGACTGGTGCCCAATCT
62	InF_XmaI_alsTfOp_rv	5′-	ATTCTCTAGACCCGGGTCATTCTTCTTCATCATCCCATTCTGGAGC
63	InF_XmaI_alsLpOp_fw	5′-	AATATAAAACCCCGGGATGCCAGACAAAAAGTATTATGGTG
64	InF_AscI_alsLpOp_rv	5′-	ATTTATTTCGGCGCGCCTTAGTAGAATTGGTCTGGCAACATAG
65	InF_SalI_aldcBsOp_fw	5′-	AACAAACAAAGTCGACATGAAGAGAGAATCCAACATCCAAG
66	InF_NotI_aldcBsOp_rv	5′-	TAAATTCACGCGGCCGCTCATTCTGGAGAACCTTCAGTAG
67	InF_SalI_aldcEaOp_fw	5′-	AACAAACAAAGTCGACATGAACCATGCTTCTGATTGCACC
68	InF_NotI_aldcEaOp_rv	5′-	TAAATTCACGCGGCCGCTTAGGATTCAACAGATCTGATGG
69	InF_SalI_aldcKpOp_fw	5′-	AACAAACAAAGTCGACATGTCCGCTTTGTTGTCTGGTG
70	InF_NotI_aldcKpOp_rv	5′-	TAAATTCACGCGGCCGCTTAGGATTCAACAGATCTGATG
71	InF_SalI_aldcLlOp_fw	5′-	AACAAACAAAGTCGACATGACCGAAATCACCCAATTATTC

72	InF_NotI_aldcLlOp_rv	5′- TAAATTCACGCGGCCGCTCATTCGGCAACGTCAATATCCTTC
73	InF_AvrII_BDH1Sc_fw	5′- CTCATATACACCTAGGATGAGAGCTTTGGCATATTT
74	InF_FseI_BDH1Sc_rv	5′- ATAAGAAATTCGCGGCCGGCCTTACTTCATTTCACCGTGAT
75	PmeI_BDH1Sc_fw	5′- ATGCGTTTAAACATGAGAGCTTTGGCATATTTCAAGAAGGGT
76	FseI_BDH1Sc_rv	5′- ATGCGGCCGGCCTTACTTCATTTCACCGTGATTGTTAGGCGT

*Underlining indicates restriction enzyme sites.
