# Supplementary material for: A pyruvate carbon flux tugging strategy for increasing 2,3-butanediol production and reducing ethanol subgeneration in the yeast Saccharomyces cerevisiae
Source: Biotechnol Biofuels. 2018 Jun 26;11:180. doi: 10.1186/s13068-018-1176-y (PMC6020211; doi:10.1186/s13068-018-1176-y)
Supplement: Supplementary file 3 — Additional file 3. Additional notes. This file includes Additional methods (Method S1–S9) and Additional results (Result S1 and S2). [file 13068_2018_1176_MOESM3_ESM.pdf]

Biotechnology for Biofuels
Additional file 3:

Additional notes (Method S1–S9, Result S1 and S2)

A pyruvate carbon flux tugging strategy for increasing 2,3-butanediol production and reducing ethanol subgeneration in the yeast *Saccharomyces cerevisiae*

Jun Ishii[a,*], Keisuke Morita[b], Kengo Ida[c], Hiroko Kato[a,b], Shohei Kinoshita[b], Shoko Hataya[a], Hiroshi Shimizu[b], Akihiko Kondo[a,c,d] and Fumio Matsuda[b,d,**]

[a] Graduate School of Science, Technology and Innovation, Kobe University, 1-1 Rokkodai, Nada, Kobe 657-8501, Japan

[b] Department of Bioinformatic Engineering, Graduate School of Information Science and Technology, Osaka University, 1-5 Yamadaoka, Suita, Osaka 565-0871, Japan

[c] Department of Chemical Science and Engineering, Graduate School of Engineering, Kobe University, 1-1 Rokkodai, Nada, Kobe 657-8501, Japan

[d] RIKEN Center for Sustainable Resource Science, 1-7-22 Suehiro, Tsurumi, Yokohama 230-0045, Japan

[**] **Corresponding author:** Fumio Matsuda
Department of Bioinformatic Engineering, Graduate School of Information Science and Technology, Osaka University, 1-5 Yamadaoka, Suita, Osaka 565-0871, Japan
Tel: +81-6-6879-7433, E-mail: fmatsuda@ist.osaka-u.ac.jp

[*] **Co-corresponding author:** Jun Ishii
Graduate School of Science, Technology and Innovation, Kobe University, 1-1 Rokkodai, Nada, Kobe 657-8501, Japan
Tel: +81-78-803-6356, E-mail: junjun@port.kobe-u.ac.jp

Additional methods

Method S1.

Yeast strains and media

Details of the yeast *S. cerevisiae* strains YPH499 [1] (Stratagene/Agilent Technologies, Palo Alto, CA, USA) and other recombinant strains used in this study and their genotypes are outlined in Table 1. Yeast-peptone-dextrose (YPDA) medium contained 10 g L^{-1} yeast extract, 20 g L^{-1} peptone, 20 g L^{-1} glucose and 40 mg L^{-1} adenine hemisulfate. Synthetic dextrose (SD) minimal medium contained 6.7 g L^{-1} yeast nitrogen base without amino acids (YNB) (BD-Diagnostic Systems, Sparks, MD, USA) and contained 20 g L^{-1} glucose as the sole carbon source. For fermentation tests, the SD medium contained a high concentration of glucose (25, 50 or 100 g L^{-1}) as the sole carbon source. Amino acids and nucleotides (40 mg L^{-1} adenine, 20 mg L^{-1} histidine, 60 mg L^{-1} leucine, 20 mg L^{-1} lysine, 40 mg L^{-1} tryptophan and 20 mg L^{-1} uracil) were appropriately supplemented as SD selection medium lacking the relevant auxotrophic components. Synthetic complete (SC) medium contained a complete range of amino acids and several additional nutrients including adenine, uracil, inositol, and *p*-aminobenzoic acid, in addition to the SD medium components.

Method S2.

Yeast strain construction

All yeast strains used in this study were generated from the YPH499 parental strain [1] and are listed in Table 1. All primers used for construction of the yeast gene deletion mutants are listed in Table S1. To generate the gene deletion mutants (Fig. S1), transformation with linear DNA fragments containing homologous recombination sequences and a *URA3* marker was performed using the lithium acetate method [2]. In accordance with the seamless marker recycling method [3], the *URA3* marker was eliminated by homologous recombination using counter selection (URA blaster) in each transformation step as depicted (Fig. S1).

The URA-blaster cassettes were constructed by the overlap PCR. For example, the cassettes for deletion of the coding DNA sequence (CDS) region of *PDC5* (1692 bp) was prepared by the overlap PCR of two fragments: i) *URA3* fragment with the 5′-untranslated (5′-UTR) region of *PDC5* (80 bp) amplified from the pGK426 plasmid [4] using the dPDC5up-URA3_fw (PDC5_URAb_1) and dPDC5up-URA3_rv (PDC5_URAb_2) primer pairs (#1 and 2) and ii) the 3′-untranslated (3′-UTR) region of *PDC5* (300 bp) with the internal homologous recombination sequence for marker pop-out (40 bp; hr40) amplified from the YPH499 genomic DNA using the dPDC5dn_fw (PDC5_URAb_3) and dPDC5dn_rv (PDC5_URAb_4) primer pairs (#3 and 4), using the primer pairs PDC5_URAb_1 and PDC5_URAb_4 (#1 and 4). Introduction of the URA-blaster cassette and pop-out of the *URA3* fragment were performed as described previously [3]. Briefly, the prepared linear DNA fragment (URA-blaster cassette) was used to transform the parental yeast strain, and the transformant was selected on solid SD medium lacking uracil. After confirming integration of the cassette at the correct locus, the cells were maintained on SC medium containing 1 mg mL^{-1} 5-fluoroorotic acid (5-FOA) (Fluorochem, Derbyshire, UK) for counter selection to elicit internal homologous recombination (pop-out) between the two hr40 sequences (the other hr40 was included in the upstream 80-bp sequence of the target gene) and eliminate the URA3 marker. Seamless and correct deletion of the target gene was confirmed by the genome PCR using the PDC5_URAb_check_fw and PDC5_URAb_check_rv primer pairs (#5 and 6) (Fig. S1).

Using the above-described procedures, a quadruple deletion mutant (*MTH1*-Δ*T* *pdc1*Δ *pdc5*Δ *pdc6*Δ) based on YPH499 as a parental strain was constructed as follows. Following the deletions of the *PDC5* and *PDC6* genes (YJI6 strain), the *MTH1*-Δ*T* allele (a 225-bp internal deletion from position 169 to 393 of the *MTH1* gene) [5] was added to the YJI6 strain, yielding the YSM009 strain (Table 1). Then, *PDC1* was deleted in the YSM009 strain, successfully resulting in the YSM021 (PDCΔ) strain (Table 1).

Method S3.

Plasmid construction and yeast transformation

All plasmids used in this study are listed in Table 2. All primers used for plasmid construction are listed in Table S1.

The plasmids used for the expression of ALSs derived from a range of organisms (16 kinds of ALS genes from 11 species (other than *S. cerevisiae*); 12 genes were from eight prokaryotes (Bs, *Bacillus subtilis*; Cg, *Corynebacterium glutamicum*; Ec, *Escherichia coli*; He, *Halomonas elongata*; Ll, *Lactococcus lactis*; Lp, *Lactobacillus plantarum*; Sg, *Streptomyces griseus*; Tf, *Thermobifida fusca*) and four genes were from three eukaryotes (Ao, *Aspergillus oryzae*; Gm, *Glycine max*; Zm, *Zea mays*); please refer to Table 2 and Fig. 2) were constructed as follows: DNA fragments encoding the ALSs were PCR-amplified from the genomic DNA of each organism using 16 sets of primer pairs (#25–56). The amplified fragments were cloned into the multiple cloning site (MCS) of the pGK425 vector [4] using the Ligation Convenience Kit (Nippon Gene, Tokyo, Japan) with appropriate restriction enzymes or the In-Fusion HD Cloning Kit (Clontech Laboratories/Takara Bio, Shiga, Japan) to yield ALS expression plasmids (Table 2). Previously constructed plasmids pGK425-ILV2 [6] and pGK425-ILV2c [7], which express yeast endogenous ALS (Ilv2) respectively in mitochondria and the cytosol, were used as the comparative ALS expression plasmids.

The high-activity ALS genes from *Lactobacillus plantarum*, *Escherichia coli* and *Thermobifida fusca* (*alsLp*, *ilvBEc* and *alsTf*) were codon optimized using GeneArt GeneOptimizer software (Life Technologies/Thermo Fisher Scientific, San Jose, CA, USA). The codon-optimized DNA fragments were ordered using the GeneArt Strings DNA fragments service (Life Technologies/Thermo Fisher Scientific) and PCR-amplified using three primer pairs (#57–62). The amplified fragments were subcloned into the MCS of the pGK425 vector using the In-Fusion HD Cloning Kit to yield codon-optimized ALS expression plasmids (Table 2). The codon-optimized *alsLp* gene (*alsLpOp*) was further PCR-amplified using a primer pair (#63 and 64) and subcloned into the MCS3 (between the *PGK1* promoter and terminator) of the pATP422 multiple gene expression vector [8] using the In-Fusion HD Cloning Kit to yield the pATP422-alsLpOp backbone plasmid for expressing multiple genes along

with *alsLpOp* (Table 2).

The plasmids used for the expression of ALDCs derived from four species (*Bacillus subtilis*, *Enterobacter aerogenes*, *Klebsiella pneumoniae* and *Lactococcus lactis*) were constructed as follows: the codon-optimized DNA fragments encoding ALDCs were ordered using the GeneArt Strings DNA fragments service and PCR-amplified using four primer pairs (#65–72). The amplified fragments were subcloned into the MCS2 (between the *TDH3* promoter and terminator) of pATP422-alsLpOp using the In-Fusion HD Cloning Kit to yield co-expression plasmids of the ALS (*alsLpOp*) and ALDC genes (Table 2).

The plasmid used for the expression of the yeast endogenous BDH was constructed as follows: the DNA fragment encoding *S. cerevisiae* BDH (*BDH1* gene) was PCR-amplified from yeast genomic DNA using a primer pair (#73 and 74). The amplified fragment was cloned into the MCS1 (between the *ADH1* promoter and terminator) of pATP422-alsLpOp using the In-Fusion HD Cloning Kit to yield the co-expression plasmid of *BDH1* genes with *alsLpOp* (pATP422-alsLpOp-BDH1) (Table 2). Similarly, the *BDH1* gene was amplified using a different primer pair (#75 and 76) and cloned into the MCS1 of pAT425 [8] using the Ligation Convenience Kit to yield the *BDH1* expression plasmid (pAT425-BDH1) (Table 2).

Transformation of plasmid DNA was carried out using the lithium acetate method [2]. YPH499 and its derivative strains (Table 1) were transformed with the yeast expression vectors or the constructed plasmids (Table 2). All transformants generated in this study are listed in Table 3.

Method S4.

Laboratory evolution

For conducting a laboratory evolution, a passage culture of the PDCΔ (YSM021) strain (Table 1) in SD medium was carried out. The cells were cultured in 5 mL of SD medium under semi-aerobic conditions, and were continuously cultivated in five independent culture series. Each independent culture series was repeatedly transferred to the new medium to give an optical density (OD) of 0.1 at 600 nm (OD_{600} = 0.1) when the cell density reached approximately OD_{600} = 0.7, and was collected to make glycerol stocks of the cells in several passages. The experiment was terminated at around 3,000 h, since further improvement was not observed. Cells of culture series 1 at days 44 and 122 (Evo44 and Evo122 strains) (Table 1) were stored as the glycerol stocks. An isolate of Evo122 (Evo122-2) was designated to be the YSM046 strain (evolved PDCΔ strain) (Table 1).

Method S5.

Genome re-sequencing

For next-generation sequencing of the whole genomes, genome DNA of YPH499, YSM021, and six evolved strains was prepared using the Qiagen Genomic-tip 100/G (Qiagen, Venlo, Netherlands). The genome libraries were sequenced by pair-end sequence (250 bp) with an Illumina HiSeq2000 using the MiSeq reagent 500 cycle kit v2 (Illumina, San Diego, CA, USA). Each sample yielded a fastq file including approximately 25 million reads, and the average quality scores per base were higher than 20. Reads were trimmed based on the quality scores, which were mapped to an S288C reference sequence obtained from the *Saccharomyces* Genome Database using Bowtie2 [9]. Variations in the genome sequence were identified using SAMtools [10]. The mapping results were confirmed using the Integrative Genomics Viewer (IGV) [11].

Method S6.

Relative activity measurement of acetolactate synthase (ALS) enzyme

Yeast cells (transformants) were grown in test tubes containing 5 mL of SD selection media at 30°C with shaking at 150 rpm for 48 h. Cells were collected, washed, and then resuspended in 400 μL of 1 M phosphate buffer (pH 7.0), and 200 μL of cell suspensions were applied to disrupt the cells using Shake Master Neo (Bio Medical Science, Tokyo, Japan) and 0.5 mm glass beads at 1,500 rpm for 5 min. Cell lysates were centrifuged at 20,000 g for 2 min, and then the supernatants (crude extracts) were collected.

The ALS activity assay followed previously described procedures [12, 13]. Relative ALS activity was determined using the colorimetric assay of acetoin (or diacetyl) by measuring the absorbance of the red color that developed. Briefly, the pre-mixture contained 25 μL of 10 mM thiamine pyrophosphate (final conc. 1 mM), 25 μL of 100 μM FAD (final conc. 10 μM), 25 μL of 100 mM $MgCl_2$ (final conc. 10 mM), 10 μL of crude extract of yeast cells (in 1 M potassium phosphate buffer at pH 7.0), and 140 μL of distilled water (total 225 μL). The 225 μL pre-mixture was pre-incubated at 30°C for 15 min, and the reaction was started by addition of 25 μL of 2 M pyruvate (final conc. 200 mM). The reaction was stopped after 20 min by addition of 35 μL of 50% (v/v) sulfonic acid and incubated at 60°C for 15 min to convert 2-acetolactate into acetoin. Acetoin was then further oxidized to diacetyl by addition of 400 μL of 0.5% (w/v) creatine and 400 μL of freshly prepared 5% (w/v) α-naphthol dissolved with 4 M NaOH. After incubation at 60°C for 15 min and cooling on ice, 100 μL of reaction solution was used to measure the absorbance at 525 nm by an EnVision multilabel plate reader (Perkin Elmer, Waltham, MA, USA).

Method S7.

Culture conditions

For fermentation tests of YPH499 transformants, (Fig. 3A and Fig. S2), yeast cells were grown in test tubes containing 3 mL of SD selection media at 30°C with shaking at 150 rpm for 48 h. Cells were collected, washed, and then resuspended in test tubes containing 3 mL of fresh SD selection media (20 g L^{-1} glucose), and fermented at 30°C with shaking at 150 rpm for 48 h under semi-aerobic conditions. For simple tuning of culture conditions and genetic modifications (Figs. 3B and 3C), the agitation speed and cover cap of the test tube were changed (150 rpm to 300 rpm, and aluminum cap to vent-type cap, SILICOSEN® (Shin-Etsu Polymer, Tokyo, Japan)), respectively.

For growth tests of the evolved PDCΔ strains (Fig. 5), yeast cells were cultured in 5 mL of SD medium at 30°C with shaking at 120 rpm. The initial OD values were set at 0.1.

For checking the culture profiles of YSM046 (evolved PDCΔ) and other yeast (YPH499 and YSM021) strains (Figs. S3, 6 and 7), yeast cells were cultured in 5 mL of SD medium at 30°C with shaking at 150 or 120 rpm. The cells were then inoculated into 5 or 100 mL of SD medium in test tubes or 500-mL Sakaguchi flasks to give an initial OD of 0.2 or 0.1, and cultured at 30°C for 24 h with shaking at 150 or 120 rpm, respectively. Test tubes containing SD selection medium were used for fermentation tests of the YSM046 and YPH499 transformants (Fig. 8).

For growth tests of PDCΔ and its evolved strains expressing *alsLpOp*, *aldcLlOp* and *BDH1* (Fig. S5), yeast cells were inoculated into 5 mL of SD selection medium to give an OD of 0.01. Then, the cells were cultured at 30°C with shaking at 70 rpm, and the OD (cell growth) was automatically monitored every 4 h using a TVS062CA biophotorecorder (Advantec Toyo, Tokyo, Japan).

For fermentations of the YHI030 (evolved PDCΔ YSM046 expressing *alsLpOp*, *aldcLlOp* and *BDH1*) strain (Figs. 9, 10, and S6–S9), yeast cells were cultured in 10 mL of SD selection medium at 30°C with shaking at 150 rpm for 3 d. A total of 7.5 mL of cell culture was transferred into 500 mL of fresh SD selection medium, and further cultivated at 30°C with shaking at 150 rpm for 3 d. After the cells were collected and washed with distilled water, 0.75 g-wet weight of cells were transferred into 50 mL of

fresh SD selection medium (containing an indicated concentration of glucose; 25, 50 or 100 g L^{-1}) to give an initial cell density of 15 g L^{-1} (wet cell weight). For buffered medium, the indicated concentration of 2-(*N*-morpholino)ethanesulfonic acid (MES) (200 or 500 mM) was added to the SD selection medium, and the pH was adjusted to 6.0. For anaerobic (oxygen-limited) conditions, small fermentation bottles with CO_2 gas outlets were used and fermentations were started at 30°C with stirring at 500 rpm. For aerobic and mild aerobic (slightly oxygen-limited) conditions, Erlenmeyer flasks were used and fermentations were started at 30°C with shaking at various agitation speeds (50–300 rpm). In Fig. S6, additional glucose (50 or 25 g L^{-1}) was added to the fermented culture (SD selection medium initially contained 50 or 25 g L^{-1} glucose) in incremental steps two times (96 and 192 h) or four times (at 48, 96, 144 and 192 h), respectively. In Fig. 10, additional glucose (100 g L^{-1}) was added to the fermented culture (initially containing 100 g L^{-1} glucose and 500 mM MES in SD selection medium) in incremental steps two times (72 and 192 h).

Method S8.

Analysis of extracellular metabolites

To determine the concentrations of 2,3-BDO, glucose, ethanol, glycerol, acetoin, pyruvate, acetate, lactate and succinate in the culture medium, supernatant obtained by centrifugation at 14,000 g for 2 min was applied to a high-performance liquid chromatography (HPLC) system (Shimadzu, Kyoto, Japan) equipped with an Aminex HPX-87H (7.8 mm, 300 mm) (Bio-Rad, Hercules, CA, USA) column, a UV/vis detector (SPD-20A; Shimadzu), and a refractive index detector (RID-10A; Shimadzu). The column temperature was set at 65°C, and 1.5 mM H_2SO_4 was used as the mobile phase with a flow rate of 0.5 mL min^{-1}. The UV detector was set at 210 nm, and the flow cell temperature of the refractive index detector was set at 40°C.

<u>Method S9.</u>

LC-MS/MS analysis of intermediate metabolites

The metabolite analysis was performed using a previously described method [14]. Briefly, 20 mL culture broth (at OD_{660} = 1.0) was sampled rapidly and filtered through a 0.5-µm pore size filter (PTFE-type membrane; Advantec, Tokyo, Japan). Cells on the filter were immediately immersed in 1.6 mL methanol (-80°C) and metabolites were extracted using the chloroform-methanol-water method. The parameters for LC-MS/MS analysis (LC: Agilent 1100 series; Agilent Technologies, Santa Clara, CA, USA) (MS/MS: API 3200; AB Sciex, Framingham, MA, USA) and selected reaction monitoring (SRM) of target metabolites were based on previously described conditions [14]. The peak of each target metabolite was identified by comparing its chromatographic behavior with that of an authentic standard. The peak area was determined using the Analyst software (version 1.6.2; AB Sciex). Quantitation was performed based on the MS data using the intracellular concentration of each metabolite (mmol g^{-1} fw^{-1}) determined using the ratio of the 13C peak area to the 12C peak area. The fresh weight (fw) was calculated as follows: (OD_{660}) × 1.50 × (sampling liquid amount).

Additional results

Result S1.

Construction of pyruvate decarboxylase-deficient (PDCΔ) strain

To disrupt the ethanol biosynthesis, we followed the previously reported pyruvate decarboxylase-deficient Pdc$^-$ strain that combined the lack of the CDS regions of the *PDC1*, *PDC5* and *PDC6* genes, and the possession of the *MTH1*-Δ*T* allele [5], because it has been demonstrated to be effective for the complete avoidance of ethanol subgeneration and the increase in 2,3-BDO production [15, 16]. We similarly constructed the pyruvate decarboxylase-deficient PDCΔ (YSM021) strain (Table 1) from YPH499 using the URA-blaster method for seamless gene deletion and marker recycling (Fig. S1) [3]. Following the deletions of the *PDC5* and *PDC6* genes, a 225-bp internal deletion from position 169 to 393 was introduced to the *MTH1* gene [5] using the URA blaster. It has been reported that Mth1p is a negative regulator of the glucose-sensing signal transduction pathway [17], and the *MTH1*-Δ*T* allele has been identified as a causal mutation in the evolved Pdc$^-$ *S. cerevisiae* strain to grow at high glucose concentrations [5]. The PDCΔ strain (YSM021) was successfully constructed by an additional *PDC1* deletion (Figs. 4A–D).

The genome sequence of the PDCΔ strain revealed that a nonsynonymous substitution (L165F) occurred in the *MTH1*-Δ*T* gene by a single nucleotide polymorphism (SNP) located at position 468 (from A to C) (Fig. 4D) (Table 1). Despite the unexpected mutation, the PDCΔ strain could grow on an SD and YPDA plate containing 20 g L^{-1} glucose as the sole carbon source (data not shown). The semi-aerobic batch cultures of the YPH499 and PDCΔ strains in 5 mL of SD medium (containing 20 g L^{-1} glucose) showed that the PDCΔ strain could grow at high glucose concentrations, although the growth rate and final OD_{660} level were lower than those of YPH499 (Fig. S3). Although glucose consumption was much slower than YPH499, no ethanol, glycerol, and acetate production was observed for PDCΔ at 120 h after the start of cultivation (Fig. S3). These profiles of the by-product formations were consistent with the previously reported *S. cerevisiae* strains lacking the *PDC1*, *PDC5* and *PDC6* genes [5, 15, 16].

Result S2.

Laboratory evolution of PDCΔ strain and genome re-sequencing

A laboratory evolution was performed to improve the slow growth rate of the PDCΔ strain. The PDCΔ (YSM021) strain was cultured in 5 mL of SD medium under semi-aerobic conditions (Fig. S4). The cells were continuously cultivated in five independent culture series that were transferred to the new medium when the cell density reached approximately OD_{600} = 0.7. The culture data showed improved growth rates for culture series 1 at approximately 800 h after the start of the experiment. The experiment using culture series 1 was terminated at day 122 (2,923 h), since further improvement was not observed (Fig. S4).

Because culture series 1 showed the fastest cell growth rate among the five culture series (Fig. S4), the glycerol stocks of the cells collected at days 44 and 122 (Evo44 and Evo122) were used for the genome re-sequencing by the next-generation sequencer. In addition to Evo122, three colonies isolated from Evo44 commonly showed improved cell growth phenotypes, indicating that causal mutations were shared among the isolated strains (Fig. 5). The genome re-sequence data revealed that five mutations were commonly found in the three isolates of Evo122 (Table 4). Among them, two nonsynonymous mutations in the *YAK1* and *MCT1* genes were also observed in the isolates from Evo44. These mutations were confirmed by the Sanger sequence of the corresponding genome regions of the Evo122-2 strain (data not shown). The *YAK1* gene (YJL141C) included an A to T mutation at position 1547 (D516V), while the *MCT1* gene (YOR221C) included a G to A mutation at position 605 (G202D) (Table 4). A predicted function of *MCT1* is a malonyl-CoA:ACP transferase responsible for lipid biosynthesis [18]. On the other hand, *YAK1* is a serine-threonine protein kinase that inhibits growth in response to glucose availability in a glucose-sensing system [19, 20], suggesting that the mutation in *YAK1* is likely responsible for the improvement in the cell growth rate. Further genetic characterization of these mutations (including L165F in the *MTH1*-Δ*T* gene) is in progress and will be reported elsewhere in the future.

Here, an isolate of Evo122 (Evo122-2) was designated to be YSM046 (Table 1) and used for the 2,3-BDO production. A comparison of the culture profiles indicated that the cell growth rate of the YSM046 strain was significantly improved from

YSM021 without production of ethanol and glycerol (Fig. S3).

References (Additional notes)

1. Sikorski RS, Hieter P. A system of shuttle vectors and yeast host strains designed for efficient manipulation of DNA in *Saccharomyces cerevisiae*. Genetics. 1989;122:19-27.
2. Gietz D, Stjean A, Woods RA, Schiestl RH. Improved method for high-efficiency transformation of intact yeast-cells. Nucleic Acids Res. 1992;20:1425-25.
3. Akada R, Kitagawa T, Kaneko S, Toyonaga D, Ito S, Kakihara Y, Hoshida H, Morimura S, Kondo A, Kida K. PCR-mediated seamless gene deletion and marker recycling in *Saccharomyces cerevisiae*. Yeast. 2006;23:399-405.
4. Ishii J, Izawa K, Matsumura S, Wakamura K, Tanino T, Tanaka T, Ogino C, Fukuda H, Kondo A. A simple and immediate method for simultaneously evaluating expression level and plasmid maintenance in yeast. J Biochem. 2009;145:701-8.
5. Oud B, Flores CL, Gancedo C, Zhang X, Trueheart J, Daran JM, Pronk JT, van Maris AJ. An internal deletion in *MTH1* enables growth on glucose of pyruvate-decarboxylase negative, non-fermentative *Saccharomyces cerevisiae*. Microb Cell Fact. 2012;11:131.
6. Kondo T, Tezuka H, Ishii J, Matsuda F, Ogino C, Kondo A. Genetic engineering to enhance the Ehrlich pathway and alter carbon flux for increased isobutanol production from glucose by *Saccharomyces cerevisiae*. J Biotechnol. 2012;159:32-7.
7. Matsuda F, Kondo T, Ida K, Tezuka H, Ishii J, Kondo A. Construction of an artificial pathway for isobutanol biosynthesis in the cytosol of *Saccharomyces cerevisiae*. Biosci Biotechnol Biochem. 2012;76:2139-41.
8. Ishii J, Kondo T, Makino H, Ogura A, Matsuda F, Kondo A. Three gene expression vector sets for concurrently expressing multiple genes in *Saccharomyces cerevisiae*. FEMS Yeast Res. 2014;14:399-411.
9. Langmead B, Salzberg SL. Fast gapped-read alignment with Bowtie 2. Nat Methods. 2012;9:357-9.
10. Li H, Handsaker B, Wysoker A, Fennell T, Ruan J, Homer N, Marth G,

Abecasis G, Durbin R, Genome Project Data Processing S. The sequence alignment/map format and SAMtools. Bioinformatics. 2009;25:2078-9.

11. Robinson JT, Thorvaldsdottir H, Winckler W, Guttman M, Lander ES, Getz G, Mesirov JP. Integrative genomics viewer. Nat Biotechnol. 2011;29:24-6.
12. Singh BK, Stidham MA, Shaner DL. Assay of acetohydroxyacid synthase. Anal Biochem. 1988;171:173-9.
13. Lee YT, Duggleby RG. Mutations in the regulatory subunit of yeast acetohydroxyacid synthase affect its activation by MgATP. Biochem J. 2006;395:331-6.
14. Nishino S, Okahashi N, Matsuda F, Shimizu H. Absolute quantitation of glycolytic intermediates reveals thermodynamic shifts in *Saccharomyces cerevisiae* strains lacking *PFK1* or *ZWF1* genes. J Biosci Bioeng. 2015;120:280-6.
15. Kim SJ, Seo SO, Jin YS, Seo JH. Production of 2,3-butanediol by engineered *Saccharomyces cerevisiae*. Bioresour Technol. 2013;146:274-81.
16. Lian JZ, Chao R, Zhao HM. Metabolic engineering of a *Saccharomyces cerevisiae* strain capable of simultaneously utilizing glucose and galactose to produce enantiopure (2R,3R)-butanediol. Metab Eng. 2014;23:92-99.
17. Lafuente MJ, Gancedo C, Jauniaux JC, Gancedo JM. Mth1 receives the signal given by the glucose sensors Snf3 and Rgt2 in *Saccharomyces cerevisiae*. Mol Microbiol. 2000;35:161-72.
18. Schneider R, Brors B, Burger F, Camrath S, Weiss H. Two genes of the putative mitochondrial fatty acid synthase in the genome of *Saccharomyces cerevisiae*. Curr Genet. 1997;32:384-8.
19. Moriya H, Shimizu-Yoshida Y, Omori A, Iwashita S, Katoh M, Sakai A. Yak1p, a DYRK family kinase, translocates to the nucleus and phosphorylates yeast Pop2p in response to a glucose signal. Genes Dev. 2001;15:1217-28.
20. Garrett S, Menold MM, Broach JR. The *Saccharomyces cerevisiae YAK1* gene encodes a protein kinase that is induced by arrest early in the cell cycle. Mol Cell Biol. 1991;11:4045-52.
